# Clarifying Associations between Childhood Adversity, Social Support, Behavioral Factors, and Mental Health, Health, and Well-Being in Adulthood: A Population-Based Study

**DOI:** 10.3389/fpsyg.2016.00727

**Published:** 2016-05-25

**Authors:** Mashhood A. Sheikh, Birgit Abelsen, Jan A. Olsen

**Affiliations:** Department of Community Medicine, University of TromsøTromsø, Norway

**Keywords:** psychological violence, emotional abuse, mental abuse, verbal aggression, verbal abuse, stress, early life stress, child maltreatment

## Abstract

Previous studies have shown that socio-demographic factors, childhood socioeconomic status (CSES), childhood traumatic experiences (CTEs), social support and behavioral factors are associated with health and well-being in adulthood. However, the relative importance of these factors for mental health, health, and well-being has not been studied. Moreover, the mechanisms by which CTEs affect mental health, health, and well-being in adulthood are not clear. Using data from a representative sample (*n* = 12,981) of the adult population in Tromsø, Norway, this study examines (i) the relative contribution of structural conditions (gender, age, CSES, psychological abuse, physical abuse, and substance abuse distress) to social support and behavioral factors in adulthood; (ii) the relative contribution of socio-demographic factors, CSES, CTEs, social support, and behavioral factors to three multi-item instruments of mental health (SCL-10), health (EQ-5D), and subjective well-being (SWLS) in adulthood; (iii) the impact of CTEs on mental health, health, and well-being in adulthood, and; (iv) the mediating role of adult social support and behavioral factors in these associations. Instrumental support (24.16%, *p* < 0.001) explained most of the variation in mental health, while gender (21.32%, *p* < 0.001) explained most of the variation in health, and emotional support (23.34%, *p* < 0.001) explained most of the variation in well-being. Psychological abuse was relatively more important for mental health (12.13%), health (7.01%), and well-being (9.09%), as compared to physical abuse, and substance abuse distress. The subjective assessment of childhood financial conditions was relatively more important for mental health (6.02%), health (10.60%), and well-being (20.60%), as compared to mother's and father's education. CTEs were relatively more important for mental health, while, CSES was relatively more important for health and well-being. Respondents exposed to all three types of CTEs had a more than two-fold increased risk of being mentally unhealthy (*RR*_Total Effect_ = 2.75, 95% CI: 2.19–3.10), an 89% increased risk of being unhealthy (*RR*_Total Effect_ = 1.89, 95% CI: 1.47–1.99), and a 42% increased risk of having a low level of well-being in adulthood (*RR*_Total Effect_ = 1.42, 95% CI: 1.29–1.52). Social support and behavioral factors mediate 11–18% (*p* < 0.01) of these effects. The study advances the theoretical understanding of how CTEs influence adult mental health, health, and well-being.

## Introduction

A significant amount of research on health and well-being has focused on assessing the influence of social support factors and behavioral factors (Armstrong, 2009). The theoretical debate in social sciences centers on the relative importance of structure and agency in determining these social support and behavioral factors. For instance, whether people's decisions about smoking, alcohol use, and making friends are shaped by structural conditions like gender, childhood socioeconomic status (CSES), childhood traumatic experiences (CTEs) etc., or if such decisions are largely a matter of agency-driven individual choices. The empirical evidence lags behind the theoretical development, and empirical evidence linking structural conditions with agency is scarce. Previous studies have shown that CSES, psychological abuse (also referred to as *psychological violence, emotional abuse, mental abuse, verbal abuse*, or exposure to *verbal aggression*) and physical abuse in childhood (also referred to as *physical violence*), and social support and behavioral factors in adulthood are associated with mental health, health, and well-being in adulthood. However, the relative contribution of these structural conditions to social support and behavioral factors, and mental health, health, and well-being has not been studied previously. We address this question by using the Shapley (Shapley, 1953) decomposition of the dissimilarity index (Hoyos and Narayan, 2011) and *R*^2^ (Huettner and Sunder, 2012) proposed by Shorrocks (1982, 2012) (see also Barros et al., 2009, 2010).

Furthermore, the mechanisms by which CTEs affect mental health, health, and well-being in adulthood are not clear. The “life course” epidemiology theory proposes the “chains of risk” model (Ben-Shlomo and Kuh, 2002), which is relevant to understanding the effect of CTEs on adult mental health, health, and well-being. Risk factors for poor health and well-being in adulthood, such as CTEs, having no social support in adulthood, smoking, and alcohol abuse, may accumulate over time as chains of risk. Each adverse experience (or exposure) tends to lead to another, and so on. In this way, different exposures or adverse experiences in life accumulate over time in an additive manner. Victims of CTEs may be more likely to encounter subsequent stressors in adulthood.

CTEs are associated with social support and behavioral factors in adulthood, including difficulties in adult interpersonal relationships and poor social conformity (Robins, 1978; Cole and Putnam, 1992; Luntz and Widom, 1994; Silverman et al., 1996; Davis et al., 2001; Horwitz et al., 2001; Schilling et al., 2007; Daruy-Filho et al., 2011; Huh et al., 2014; Krastins et al., 2014; Gayer-Anderson et al., 2015), increased risk of higher alcohol use (Miller et al., 1993; Widom et al., 1995; Fergusson and Lynskey, 1997; McCauley et al., 1997; Widom and White, 1997; Felitti Md et al., 1998; Hussey et al., 2006; Shin et al., 2015), smoking (Felitti Md et al., 1998; Hussey et al., 2006), and a wide range of mental health problems (Heim and Binder, 2012; Norman et al., 2012; Gilman et al., 2015), which may also affect health negatively (McLaughlin et al., 2010; Shonkoff and Garner, 2011). Thus, social support and behavioral factors in adulthood shape later health and well-being, but they are also influenced by antecedent conditions (Schilling and Christian, 2014). This implies that disadvantages in health that are associated with social support and behavioral factors in adulthood may be contingent upon the structural situations that provoked and shaped these factors in the first place.

When the results of previous studies on the role of social support factors in adulthood as mediators in the CTEs-health association are considered, the dominant conclusion is that victims of CTEs may display antisocial behavior (or may have developed an antisocial personality disorder) as a consequence of CTEs. *The Diagnostic and Statistical Manual of Mental Disorders* (5th edn; DSM–5) describes abuse during childhood as one of the predisposing factors for antisocial personality disorder (American Psychiatric Association, 2013). Thus, it may not be the *lack* of social support network that mediates the CTEs -health/well-being association; rather, it may be the *incapacity* to maintain a social support network, which in turn affects health and well-being negatively. In this way, social support factors in adulthood may serve as a crude proxy for antisocial behaviors or disorders. This raises an important, yet rarely addressed question: is the influence of CTEs on adult health and well-being independent of social support and behavioral risk factors in adulthood? These associations are probabilistic rather than deterministic and the chain of risk may be broken by intervening on the mediators, but a residual damage may remain in the form of direct effects. Only a few studies (Shaw and Krause, 2002; Dong et al., 2003; Springer, 2009; Morton et al., 2014; Salinas-Miranda et al., 2015) have assessed the mediating role of social support factors and behavioral factors in the CTEs-health association, and the results were not consistent. Behavioral factors, such as smoking and a higher alcohol use, may serve as coping mechanisms or as self-medication for victims of CTEs, leading to increased health risks in adulthood (Briere, 2002; Morton et al., 2014).

Several studies (Felitti Md et al., 1998; Dube et al., 2001; Edwards et al., 2003; Schilling et al., 2007; Hovens et al., 2010; Raposo et al., 2014) have assessed the effect of childhood adversity on adult outcomes by assigning a score constructed by counting the stressors that occurred. However, this approach assumes that each type of CTE has an equivalent weight, and that there is an additive effect, when in fact some CTEs may have a stronger effect than others, and there may not be any additive effect (Cohen et al., 1997; Martin et al., 2006; Schilling et al., 2008). For instance, previous studies have shown that psychological abuse has a greater negative effect on mental health and health in adulthood, as compared to physical abuse in childhood (Ney, 1987; Martin et al., 2006; Norman et al., 2012; Dias et al., 2014; Spinazzola et al., 2014; Auslander et al., 2015; Friborg et al., 2015).

Furthermore, are the effects of different adversities in childhood distinct from one another? Since different adversities may be correlated, interact, and co-occur in the same individuals, is there an independent and unique effect of each indicator of CTEs on adult mental health, health, and well-being? Only a few studies (Mullen et al., 1996; Greenfield and Marks, 2009, 2010; Slopen et al., 2010; Dias et al., 2014; Thoresen et al., 2015) have considered (though it was not explicitly stated in most of them) the inter-dependence (multiplicative interaction) between different types of CTEs, and the results were not consistent. This may be because the low prevalence of CTEs make interactions difficult to detect in small samples.

Many studies have relied on high-risk samples, treatment-seeking samples, diagnosed patient samples, and reported cases (Alloy et al., 2006; Gaudiano and Zimmerman, 2010; Saunders and Adams, 2014; Cancel et al., 2015; van Dam et al., 2015). These samples are more prone to selection bias, and are not helpful in making population estimates (Chartier et al., 2010; Saunders and Adams, 2014). It is difficult to establish whether social support and behavioral factors in adulthood in general are involved in the etiology of health outcomes from these samples.

The influence of CTEs extends to single-item self-rated health (Felitti Md et al., 1998; Hussey et al., 2006; Fagundes and Way, 2014; Salinas-Miranda et al., 2015) and psychological well-being (Greenfield and Marks, 2010; Nurius et al., 2015) in adulthood. Previous studies have shown that the single-item, self-rated health questions are an unreliable measure of health (Crossley and Kennedy, 2002; Zajacova and Dowd, 2011), in contrast to disease-specific or symptom-specific measures of health (Sheikh et al., 2016). Similarly, over 70% of the variation in the single-item global life satisfaction question is driven by the mood we are in at the very moment we are asked the question (Seligman, 2012).

Few previous studies (Felitti Md et al., 1998; Walker et al., 1999; Edwards et al., 2003; Agorastos et al., 2014) have assessed the association between CTEs and quality of life in adulthood. However, no previous study was found that assessed the influence of CTEs on a validated generic descriptive system for health-related quality of life (HRQoL) such as the Euroqol 5 dimension scale (EQ-5D), or subjective well-being (SWLS) in adulthood.

## Aims of the study

In this study, we assessed (i) The relative contribution of structural conditions (gender, age, CSES, psychological abuse, physical abuse, and substance abuse distress) to social support and behavioral factors in adulthood; (ii) the relative contribution of socio-demographic factors, CSES, CTEs, social support, and behavioral factors to three multi-item instruments of mental health (SCL-10), health (EQ-5D), and subjective well-being (SWLS) in adulthood; (iii) the impact of CTEs on mental health, health, and well-being in adulthood, and; (iv) the mediating role of adult social support and behavioral factors in these associations.

## Data and methods

### Study population

Tromsø is the largest city in Northern Norway, with more than 70,000 inhabitants. The Tromsø Study is a prospective cohort study of the population residing in the municipality of Tromsø that is considered representative of the adult population there (Jacobsen et al., 2012). Between 1974 and 2007/2008, six waves of the Tromsø Study were conducted (referred to as Tromsø I–VI). The current paper is based on data from the sixth wave, conducted in 2007/2008. For this wave 19,762 subjects were invited; 12,984 (65.7%) returned the questionnaire (6054 men and 6930 women, born between 1920 and 1977). The study design has been described previously in detail (Jacobsen et al., 2012).

### Measures of mental health, health, and well-being

Mental health status was measured by the Hopkins Symptoms Check List-10 (SCL-10), which is widely used in epidemiological studies. Respondents rated each of the 10 items in the SCL-10 on a four-point scale ranging from not at all (1) to extremely (4). We found an acceptable degree of internal consistency for the four-point scale in this sample (Cronbach's alpha: 0.87, mean inter-item correlation: 0.43, McDonald's omega coefficient for composite reliability: 0.939).

The average SCL-10 score was calculated by dividing the total score by the total number of items (range: 1.0–4.0) (Strand et al., 2003). An SCL-10 score of 1.85 has been proposed as the cut-off for predicting diagnosed mental disorders (Strand et al., 2003) and was used in this study. A composite binary mental health status variable was constructed by classifying respondents with scores below 1.85 as mentally healthy (*Y* = 0), and those with scores ≥1.85 as mentally unhealthy (*Y* = 1). In addition to the binary variable, a separate continuous variable was constructed as the sum of the 10 items. The total sum of scores were linearly transformed from 0 to 1, where 1 represents the worst mental health, and 0 represents perfect mental health (mean: 0.09, SD: 0.13), to facilitate comparison between the three measures of mental health, health, and well-being.

Health was assessed in the study questionnaire by the EQ-5D generic measure of health-related quality of life. The EQ-5D includes five health dimensions: mobility, self-care, usual activities, pain/discomfort, and anxiety/depression (The EuroQol Group., 1990). Each health dimension has three levels: (1) no problems, (2) some problems, and (3) unable or extreme problems. The sum of five indicators (range: 5–13) was divided in three groups (tertiles), with score ranges: 5 (healthy), 6 slightly unhealthy; implying one level down to “some problems” on only one of the five dimensions, and 7–13 (unhealthy). Those with the scores 7–13 were classified as unhealthy (*Y* = 1), while those with the scores 5–6 were classified as relatively healthy (*Y* = 0). In addition to the binary variable, a separate continuous variable was constructed as the sum of the five items. The total sum of scores were then linearly transformed from 0 to 1; where 1 represents the worst health, and 0 represents perfect health (mean: 0.10, SD: 0.12).

Well-being was measured by the response to the first three items on the satisfaction with life scale (SWLS; Diener et al., 1985). These were: “In most ways my life is close to my ideal,” “The conditions of my life are excellent,” and “I am satisfied with my life.” Respondents rated these statements using a 7-point scale ranging from completely disagree (1) to completely agree (7). The sum of three indicators (range: 3–21) was divided in three groups (tertiles), with score ranges: 3–15 (low level of well-being), 16–18 (neither low nor high well-being), and 19–21 (high level of well-being). Those with scores 3–15 were classified as having a low level of well-being (*Y* = 1), while those with the scores 16–21 were classified as having a relatively high level of well-being (*Y* = 0). In addition to the binary variable, a separate continuous variable was constructed as the sum of the three items. The scores were inverted, so that a higher score represents lower well-being. The total sum of scores was linearly transformed from 0 to 1; where 1 represents the lowest well-being, and 0 represents the highest well-being (mean: 0.27, SD: 0.20).

The binary variables of mental health, health, and well-being were used for analyses with Shapley decomposition, chi-square tests, and Poisson regression models. The continuous variables (scale: 0–1) were used for analyses with Shapley decomposition, quantile regression models, and analysis of variance [ANOVA with F^*^ tests and Welch (*W*) tests]. In addition, we performed all analysis with alternative cut-offs (see Online Supplementary Material).

### Childhood traumatic experiences (CTEs)

Self-reported information on CTEs was collected by the question: “Have you over a long period experienced any of the following? (as a child),” followed by three types of traumatic experiences: (i) Being tormented, or threatened with violence; (ii) Being beaten, kicked, or the victim of other types of violence, and; (iii) Someone in your close family using alcohol or drugs in such a way that caused you worry. Respondents who ticked one or more of these responses were classified as exposed to psychological abuse, physical abuse, and substance abuse distress, respectively. To assess whether there is an additive effect of CTEs on mental health, health, and well-being, we constructed a separate variable of *trauma frequency*: 0 = not exposed to any CTE (reference), 1 = exposed to any one CTE, 2 = exposed to any two CTEs, 3 = exposed to all three CTEs.

### Mediators

Social support and behavioral factors in adulthood were used as mediators. Social support was measured with indicators of instrumental/tangible support and emotional support. Instrumental or tangible support was measured as: “Do you have enough friends who can give you help and support when you need it?” (yes = 0, no = 1). Emotional support was measured as: “Do you have enough friends you can talk confidentially with?” (yes = 0, no = 1). Behavioral factors were measured as: “Do you/did you smoke daily?” (yes, currently; yes, previously; never (reference)); and “How many units of alcohol (a beer, a glass of wine, or other alcoholic beverage) do you usually drink when you drink alcohol?” 1 = 1–4, 2 = 5–6, 3 = 7–9, 4 = 10 or more).

### Socio-demographic factors and childhood socioeconomic status (confounding variables)

The potential confounding variables: gender (0 = female, 1 = male), age (range: 30–87, mean: 57.52, standard deviation: 12.66, standard error: 0.11), mother's education, father's education, and childhood financial conditions, were chosen based on *a priori* knowledge of the association between the exposures, mediators and outcomes under study (Hernán et al., 2002). Three indicators of CSES were used in this study; mother's education, father's education, and subjective assessment of childhood financial conditions. Respondents reported their mother's and father's education separately as: 1 = primary and secondary school or similar (7–10 years of schooling), 2 = vocational school, 3 = high school, 4 = college or university (less than 4 years), and 5 = college or university (4 years or more). The variable childhood financial conditions was measured retrospectively by the question: “How was your family's financial situation when you were a child?” on a 4-point scale (1 = very good, 2 = good, 3 = difficult, 4 = very difficult).

### Statistical analyses

All analyses were performed using Stata version 13. The pattern of missingness was arbitrary in the dataset (data not shown). Assuming that the data is missing at random, 100 duplicate datasets were generated with multiple imputation (MI), to avoid sampling variability due to random iterations (StataCorp., 2013). In order to increase the predictive power of the imputation procedure, we included all indicators of socioeconomic status, mental health, health, and well-being in imputation models. A comparison between the complete-case and the imputed dataset is presented with proportions (%) within each category of the variables (Tables 1, 2). Mean (standard error), median, and proportion of respondents in each category was calculated in the complete-case dataset and in the imputed dataset with MI (Tables 1, 2).

**Table 1:** **General characteristics of the study sample in the complete-case dataset and the imputed dataset (*N* = 12,981)**.

**Characteristics**		**Complete-case dataset**		**Imputed dataset**
		***n*^a^**	**%**	**%**
Gender	Female	6928	53.4	–^b^
	Male	6053	46.6	–^b^
Age	mean (std. err)	51.52 (0.11)		
	median	59		
	30–39	509	3.9	–^b^
	40–49	3574	27.5	–^b^
	50–59	2436	18.8	–^b^
	60–69	4102	31.6	–^b^
	70–79	1829	14.1	–^b^
	80–89	531	4.1	–^b^
Mother's education^a^	mean (std. err)	1.37(0.01)		1.37 (0.01)
	median	1		1
	Primary and secondary school or similar (7–10 years)	9233	78.7	79.0
	Vocational school	1473	12.6	12.3
	High school	338	2.9	2.8
	College or university (< 4 years)	500	4.3	4.2
	College or university (≥4 years)	185	1.6	1.6
Father's education^a^	mean (std. err)	1.65 (0.01)		1.64 (0.01)
	median	1		1
	Primary and secondary school or similar (7–10 years)	7435	64.2	64.6
	Vocational school	2480	21.4	21.2
	High school	427	3.7	3.6
	College or university (< 4 years)	731	6.3	6.1
	College or university (≥4)	507	4.4	4.3
Childhood financial conditions^a^	mean (std. err)	2.23 (0.01)		2.23 (0.01)
	median	2		2
	Very good	699	5.8	5.8
	Good	8011	66.6	66.6
	Difficult	3113	25.9	25.9
	Very difficult	204	1.7	1.6
Childhood traumatic experiences	No traumatic experience (Ps_0_Ph_0_D_0_)	10,907	84.0	–^*b*^
	Psychological abuse only (Ps_1_Ph_0_D_0_)	525	4.0	–^b^
	Physical abuse only (Ps_0_Ph_1_D_0_)	230	1.8	–^b^
	Substance abuse distress only (Ps_0_Ph_0_D_1_)	643	4.9	–^b^
	Psychological and physical abuse (Ps_1_Ph_1_D_0_)	393	3.0	–^b^
	Psychological abuse and substance abuse distress (Ps_1_Ph_0_D_1_)	106	0.8	–^b^
	Physical abuse and substance abuse distress (Ps_0_Ph_1_D_1_)	44	0.3	–^b^
	Psychological abuse, physical abuse, and substance abuse distress (Ps_1_Ph_1_D_1_)	133	1.0	–^b^

*Ps_0_Ph_0_D_0_: Not exposed to psychological abuse, physical abuse and substance abuse distress in childhood. Ps_1_Ph_0_D_0_: Exposed to psychological abuse but not physical abuse and substance abuse distress. Ps_0_Ph_1_D_0_: Exposed to physical abuse but not psychological abuse and substance abuse distress. Ps_0_Ph_0_D_1_: Exposed to substance abuse distress, but not psychological abuse and physical abuse. Ps_1_Ph_1_D_0_: Exposed to both psychological and physical abuse but not substance abuse distress. Ps_1_Ph_0_D_1_: Exposed to both psychological abuse and substance abuse distress but not physical abuse. Ps_0_Ph_1_D_1_: Exposed to both physical abuse and substance abuse distress but not psychological abuse. Ps_1_Ph_1_D_1_: Exposed to psychological abuse, physical abuse, and substance abuse distress*.

a *The numbers do not add up to 12981 due to missing values*.

b *There were no missing values, so no imputations were made for these variables*.

**Table 2:** **Proportion (%) of the mediating factors and health and well-being in the complete-case dataset, and in the imputed dataset with multiple imputation (*N* = 12,981)**.

		**Complete-case dataset %**	**Imputed dataset %**
**MEDIATORS**			
Instrumental support^a^	Yes	90.6	88.8
	No	9.4	11.2
Emotional support^a^	Yes	88.1	87.1
	No	11.9	12.9
Daily smoking ^b^	Never	36.6	37.3
	Previous smoker	43.1	42.3
	Current smoker	20.3	20.4
Alcohol use (units)^b^	1–4	89.8	90.9
	5–6	7.4	6.8
	7–9	2.1	1.8
	10 or more	0.6	0.6
**MENTAL HEALTH, HEALTH, AND WELL-BEING**			
Mental health (SCL-10)	Healthy	91.9	90.7
	Unhealthy	8.1	9.3
	mean (std. err)	0.09 (0.001)	0.10 (0.001)
Health (EQ-5D)	Healthy	73.7	72.6
	Unhealthy	26.3	27.4
	mean (std. err)	0.09 (0.001)	0.11 (0.001)
Well-being (SWLS)	High	62.5	62.3
	Low	37.6	37.7
	mean (std. err)	0.27 (0.002)	0.28 (0.002)

a *Social support factors were measured by instrumental and emotional support. Instrumental support: Do you have enough friends who can give you help and support when you need it? (yes, no); Emotional support: Do you have enough friends you can talk confidentially with? (yes, no)*.

b *Behavioral factors were measured by two questions: Do you/did you smoke daily? (yes, now; yes, previously; never); How many units of alcohol (a beer, a glass of wine or a drink) do you usually drink when you drink alcohol? (1–4, 5–6, 7–9, 10, or more)*.

*SCL-10: Mental health status was measured by the Hopkins Symptoms Check List-10 (SCL-10). EQ-5D: Health was assessed by the EQ-5D generic measure of health-related quality of life. SWLS: Well-being was measured by the satisfaction with life scale (SWLS)*.

### Assessing the relative contribution of socio-demographic factors, childhood socioeconomic status and childhood traumatic experiences to social support and behavioral factors in adulthood

We used Shapley (1953) decomposition of the dissimilarity index (Hoyos and Narayan, 2011) and *R*^2^ (Huettner and Sunder, 2012) proposed by Shorrocks (1982, 2012), to examine the relative importance of socio-demographic factors, CSES and CTEs to social support and behavioral factors in adulthood (Table 3).

**Table 3:** **Relative contribution of structural conditions to social support and behavioral factors in adulthood**.

	**Shapley decomposition (% explained)**			
	**Instrumental support**	**Emotional support**	**Alcohol use**	**Smoking**
**Explanatory variables**	**%**	**%**	**%**	**%**
Gender	0.17	35.73^d^	41.18^d^	9.75^d^
Age	22.82^d^	8.26^d^	53.19^d^	1.76^b^
Mother's education	1.49	0.30	0.72^d^	27.01^d^
Father's education	1.24^d^	0.86^c^	0.48	32.52^d^
Childhood financial conditions	40.50^d^	29.26^d^	0.83^b^	7.83^b^
Psychological abuse	20.04^d^	14.06^d^	0.30	1.98^b^
Physical abuse	8.72^a^	8.35^a^	0.90^b^	15.90^d^
Substance abuse distress	5.01^c^	3.17	2.39^d^	3.26^a^

a *P < 0.1*.

b *P < 0.05*.

c *P < 0.01*.

d *P < 0.001*.

*Instrumental support: Do you have enough friends who can give you help and support when you need it? (yes, no)*.

*Emotional support: Do you have enough friends you can talk confidentially with? (yes, no)*.

*Alcohol use: How many units of alcohol (a beer, a glass of wine or a drink) do you usually drink when you drink alcohol? (1–4, 5–6, 7–9, 10 or more)*.

*Smoking: Do you/did you smoke daily? [yes, now; yes, previously; never (ref)]*.

### Assessing the relative contribution of socio-demographic factors, childhood socioeconomic status, childhood traumatic experiences, social support, and behavioral factors to adult mental health, health, and well-being

We used the Shapley decomposition of dissimilarity index (Shorrocks, 1982, 2012; Hoyos and Narayan, 2011) to examine the relative importance of socio-demographic factors, CSES, CTEs, social support, and behavioral factors to mental health, health, and well-being in adulthood. The Shapley decomposition is based on the Shapley value concept in cooperative games to distribute among the players the surplus produced by a coalition of players among those players. The Shapley decomposition represents the extent to which an outcome varies (thereby, the inequality in mental health, health, or well-being) when a predictor is added to different pre-existing sets of predictors. The change in marginal probability of the outcome after adding a predictor gives the proportion of contribution influenced by that predictor. However, since the predictors may be correlated, the change in outcome obtained by adding a predictor depends on the initial set of predictors to which it was added. Therefore, to measure the relative contribution of a predictor (***x***), the Shapley decomposition takes the average of all marginal impacts when the predictor ***x*** is added to all possible subsets of all other predictors considered. The total proportion (100%) is then divided among the predictors based on their average marginal impacts (Tables 4, 5).

**Table 4:** **Relative contribution of socio-demographic factors, childhood socioeconomic status, childhood traumatic experiences, social support and behavioral factors for mental health (SCL-10), health (EQ-5D), and subjective well-being (SWLS)**.

	**Shapley decomposition of dissimilarity index (% explained)**		
	**Mental health (SCL-10)**	**Health (EQ-5D)**	**Well-being (SWLS)**
**Explanatory variables**	**%**	**%**	**%**
Gender	10.81^d^	21.32^d^	2.33^d^
Age	2.56^c^	11.08^d^	1.01
Mother's education	0.93^d^	4.44^d^	2.27^c^
Father's education	0.61^c^	2.68	1.30^a^
Childhood financial conditions	6.02^d^	10.60^d^	20.60^d^
Psychological abuse	12.13^d^	7.01^d^	9.09^d^
Physical abuse	5.30^d^	4.19^c^	3.63
Substance abuse distress	2.73	1.72	4.72^b^
Instrumental support^a^	24.16^d^	12.02^d^	19.95^d^
Emotional support^a^	20.62^d^	10.87^d^	23.34^d^
Alcohol use (units)^b^	4.82^d^	2.21^d^	4.31^d^
*Daily smoking*^b^			
Never smoker (ref.)	Ref.	Ref.	Ref.
Previous smoker	0.85^d^	2.34^d^	0.55^c^
Current smoker	8.46^d^	9.50^d^	6.88^d^
Human Opportunity Index	0.05	0.19	0.31
Dissimilarity index	0.33	0.20	0.13
Penalty	0.02	0.05	0.05
Coverage	0.07	0.24	0.36

a *P < 0.1*.

b *P < 0.05*.

c *P < 0.01*.

d *P < 0.001*.

*^a^Social support factors were measured by instrumental and emotional support. Instrumental support: Do you have enough friends who can give you help and support when you need it? (yes, no); Emotional support: Do you have enough friends you can talk confidentially with? (yes, no)*.

*^b^Behavioral factors were measured by two questions: Do you/did you smoke daily? [yes, now; yes, previously; never (ref)]; How many units of alcohol (a beer, a glass of wine or a drink) do you usually drink when you drink alcohol? (1–4, 5–6, 7–9, 10 or more)*.

*SCL-10: Mental health status was measured by the Hopkins Symptoms Check List-10 (SCL-10)*.

*EQ-5D: Health was assessed by the EQ-5D generic measure of health-related quality of life*.

*SWLS: Well-being was measured by the satisfaction with life scale (SWLS)*.

**Table 5:** **Relative contribution of socio-demographic factors, childhood socioeconomic status, childhood traumatic experiences, social support, behavioral factors and mental health to health (EQ-5D), and subjective well-being (SWLS)**.

	**Shapley decomposition of** ***R***^**2**^**(% explained)**	
	**Health (EQ-5D)^g^**	**Well-being (SWLS)^h^**
**Explanatory group of variables**	**%**	**%**
Socio-demographic factors^a^	8.45	0.54
Childhood socioeconomic status^b^	4.06	5.07
Childhood traumatic experiences^c^	3.31	3.29
Social support factors^d^	5.35	15.25
Behavioral factors^e^	3.22	1.63
Mental health^f^	75.61	51.33
Health^g^	–	22.89
Model *R*^*2*^	0.30	0.25
*F*	253.79	171.73
*p*	< 0.001	< 0.001

a *Gender and age*.

b *Mother's education, father's education and childhood financial conditions*.

c *Psychological abuse, physical abuse and substance abuse distress*.

d *Social support factors were measured by instrumental and emotional support. Instrumental support: Do you have enough friends who can give you help and support when you need it? (yes, no); Emotional support: Do you have enough friends you can talk confidentially with? (yes, no)*.

e *Behavioral factors were measured by two questions: Do you/did you smoke daily? (yes, now; yes, previously; never (ref)); How many units of alcohol (a beer, a glass of wine or a drink) do you usually drink when you drink alcohol? (1–4, 5–6, 7–9, 10 or more)*.

f *SCL-10: Mental health status was measured by the Hopkins Symptoms Check List-10 (SCL-10); scale (0–1), where 0 represents perfect mental health, and 1 represents worst mental health*.

g *EQ-5D: Health was assessed by the EQ-5D generic measure of health-related quality of life; scale (0–1), where 0 represents perfect health, and 1 represents worst health*.

h *SWLS: Well-being was measured by the satisfaction with life scale (SWLS) ; scale (0–1), where 0 represents highest well-being, and 1 represents lowest well-being*.

### Assessing the relative contribution of socio-demographic factors, childhood socioeconomic status, childhood traumatic experiences, social support, behavioral factors and mental health to health, and well-being

To assess the relative importance of mental health to health, and that of mental health and health to well-being, we used Shapley decomposition of *R*^2^ (Table 5). The continuous variables (scale: 0–1) of mental health, health, and well-being were used (Table 5).

### Independent influence of each explanatory variable on mental health, health, and well-being

The association between all the explanatory variables, and mental health, health, and well-being [continuous outcomes (scale: 0–1)] was assessed with quantile regression models (Table 6). All independent variables used in this study were included as predictors together in the model (adjusted for each other). Therefore, the estimates presented in Table 6 present the independent influence of each predictor (Table 6). In contrast to the ordinary least square (OLS) model, quantile regression uses the conditional median function *Q*_*q*_*(y|x*_*i*…*k*_*)*, where median is the 50th percentile. The quantile *q* ∈ *(0, 1)* is that *y* splits the data into proportions *q* below and *1* − *q* above: *F(y*_*q*_*)* = *q* and *y*_*q*_ = *F* ^−1^ *(q)*: for the median, *q* = 0.5. The Huber sandwich estimator was used for the variance-covariance matrix, which does not assume that the errors are independently and identically distributed. The quantile regression model minimizes model prediction error ∑i |e_i_|, in contrast to ∑i |e${}_{\text{i}}^{2}$|in the OLS model, and is therefore more robust in terms of deviation from a parametric distribution of errors.

**Table 6:** **Independent association between all explanatory variables, and mental health (SCL-10), health (EQ-5d), and subjective well-being (SWLS) with quantile regression model (*N* = 12,981)**.

	**Mental health (SCL-10)**	**Health (EQ-5D)**	**Well-being (SWLS)**
**Explanatory variables**	**β (95% CI)**	**β (95% CI)**	**β (95% CI)**
Gender	**–0.049 (–0.059 to –0.040)**^d^	**–0.055 (–0.060 to –0.051)**^d^	**–0.014 (–0.022 to –0.005)**^c^
Age	0.000 (–0.000 to 0.000)	**0.001 (0.001–0.001)**^d^	**–0.001 (–0.001 to 0.000)**^c^
Mother's education	–0.000 (–0.004 to 0.003)	**–0.003 (–0.005 to –0.002)**^d^	**–0.008 (–0.012 to –0.004)**^*d*^
Father's education	0.000 (–0.003 to 0.003)	–0.001 (–0.002–0.000)	**0.005 (0.001–0.009)**^b^
Childhood financial conditions	**0.017 (0.012–0.021)**^d^	**0.018 (0.015–0.022)**^d^	**0.049 (0.043–0.056)**^d^
No trauma (Ps_0_Ph_0_D_0_)	*ref*	*ref*	*ref*
Psychological abuse only (Ps_1_Ph_0_D_0_)	**0.050 (0.036–0.063)**^d^	**0.030 (0.017–0.043)**^d^	**0.041 (0.024–0.058)**^d^
Physical abuse only (Ps_0_Ph_1_D_0_)	**0.025 (–0.000 to 0.051)***^a^*	0.018 (–0.004 to 0.039)	**0.048 (0.026–0.069)**^d^
Substance abuse distress only (Ps_0_Ph_0_D_1_)	**0.017 (0.005–0.028)**^c^	0.002 (–0.006 to 0.010)	**0.035 (0.026–0.045)**^d^
Psychological and physical abuse (Ps_1_Ph_1_D_0_)	**0.056 (0.032–0.080)**^d^	**0.042 (0.035 to 0.049)**^d^	**0.070 (0.055–0.084)**^d^
Psychological abuse and substance abuse distress (Ps_1_Ph_0_D_1_)	**0.094 (0.053–0.135)**^d^	**0.040 (0.016–0.065)**^d^	**0.101 (0.058–0.143)**^d^
Physical abuse and substance abuse distress (Ps_0_Ph_1_D_1_)	0.021 (–0.022 to 0.064)	0.035 (–0.008 to 0.077)	0.005 (–0.053 to 0.063)
Psychological abuse, physical abuse and substance abuse distress (Ps_1_Ph_1_D_1_)	**0.084 (0.061–0.106)**^d^	**0.051 (0.042–0.061)**^d^	**0.062 (0.019–0.105)**^c^
Instrumental support^e^	**0.068 (0.053–0.082)**^d^	**0.062 (0.052–0.073)**^d^	**0.110 (0.091–0.129)**^d^
Emotional support^e^	**0.049 (0.039–0.061)**^d^	**0.024 (0.014–0.034)**^d^	**0.088 (0.071–0.106)**^d^
Alcohol use (units)^f^	**0.016 (0.008–0.025)**^d^	**0.019 (0.011–0.026)**^d^	**0.024 (0.014–0.035)**^d^
*Daily smoking*^f^	–	–	–
Never smoker (ref)	*ref*	*ref*	*ref*
Past smoker	**0.016 (0.007–0.025)**^d^	**0.012 (0.008–0.015)**^d^	**0.017 (0.007–0.027)**^c^
Daily smoker	**0.019 (0.005–0.033)**^c^	**0.028 (0.023–0.034)**^d^	**0.039 (0.029–0.048)**^d^

a *P < 0.1*.

b *P < 0.05*.

c *P < 0.01*.

d *P < 0.001*.

e *Social support factors were measured by instrumental and emotional support. Instrumental support: Do you have enough friends who can give you help and support when you need it? (yes, no); Emotional support: Do you have enough friends you can talk confidentially with? (yes, no)*.

f *Behavioral factors were measured by two questions: Do you/did you smoke daily? [yes, now; yes, previously; never (ref)]; How many units of alcohol (a beer, a glass of wine or a drink) do you usually drink when you drink alcohol? (1–4, 5–6, 7–9, 10 or more)*.

*SCL-10: Mental health status was measured by the Hopkins Symptoms Check List-10 (SCL-10); scale (0–1), where 0 represents perfect mental health, and 1 represents worst mental health*.

*EQ-5D: Health was assessed by the EQ-5D generic measure of health-related quality of life; scale (0–1), where 0 represents perfect health, and 1 represents worst health*.

*SWLS: Well-being was measured by the satisfaction with life scale (SWLS); scale (0–1), where 0 represents highest well-being, and 1 represents lowest well-being*.

*^g^ All variables are adjusted for each other*.

*All significant associations are in bold*.

### Association between childhood traumatic experiences, and social support and behavioral factors in adulthood

The association between the different combinations of CTEs, and social support and behavioral factors was assessed with cross-tabulation with chi-square tests, F^*^ test and Welch (*W*) test (Table 7).

**Table 7:** **Distribution (%) of mediators and measures of health and well-being by exposure to childhood traumatic experiences**.

		**Ps_0_Ph_0_D_0_*n* = 10, 907**	**Ps_1_Ph_0_D_0_*n* = 525**	**Ps_0_Ph_1_D_0_*n* = 230**	**Ps_0_Ph_0_D_1_*n* = 643**	**Ps_1_Ph_1_D_0_*n* = 393**	**Ps_1_Ph_0_D_1_*n* = 106**	**Ps_0_Ph_1_D_1_*n* = 44**	**Ps_1_Ph_1_D_1_*n* = 133**	**Test statistic**	***p***
**MEDIATORS**											
Instrumental support^a^	Yes	90.1	82.4	85.8	86.5	76.8	79.8	88.4	84.1	$\chi_{\left(7\right)}^{2}=$ 110.02	*p* < 0.001
	No	9.9	17.6	14.2	13.5	23.2	20.2	11.6	15.9		
Emotional support^a^	Yes	88.4	81.0	81.9	84.7	74.7	81.7	88.6	80.2	$\chi_{\left(7\right)}^{2}=$ 100.88	*p* < 0.001
	No	11.6	19.0	18.1	15.3	25.3	18.3	11.4	19.8		
Daily smoking^b^	Never	37.7	39.3	31.1	36.1	34.2	35.6	27.9	25.8	$\chi_{\left(14\right)}^{2}=$ 27.38	*p* < 0.05
	Previously	42.1	42.6	45.8	43.4	43.4	33.7	41.9	47.7		
	Yes	20.2	18.1	23.1	20.5	22.4	30.8	30.2	26.5		
Alcohol units^b^, ^c^	1–4	91.1	90.1	84.5	87.3	83.3	81.5	78.0	83.1	$\chi_{\left(21\right)}^{2}=$ 80.40	*p* < 0.001
	5–6	6.7	6.7	11.1	9.2	11.1	14.1	12.2	14.5		
	7–9	1.8	2.3	3.9	2.6	3.6	2.2	7.3	1.6		
	10 or more	0.5	0.8	0.5	0.9	1.9	2.2	2.4	0.8		
	mean	1.12	1.14	1.20	1.17	1.24	1.25	1.34	1.20	F*_(7, 506.89)_ = 5.38 *W*_(7, 382.76)_ = 5.26	*p* < 0.001
**MENTAL HEALTH, HEALTH, AND WELL-BEING**											
Mental health (SCL-10)	Healthy	93.1	84.1	92.3	90.4	82.2	77.4	86.8	73.5	$\chi_{\left(7\right)}^{2}=$ 179.49	*p* < 0.001
	Unhealthy	6.9	15.9	7.7	9.6	17.8	22.6	13.2	26		
	mean	0.08	0.15	0.11	0.11	0.16	0.21	0.12	0.20	F*_(7, 775.81)_ = 36.62 *W*_(7, 363.64)_ = 33.45	*p* < 0.001
Health (EQ-5D)	Healthy	75.5	63.9	67.7	72.7	61.3	55.3	67.4	54.6	$\chi_{\left(7\right)}^{2}=$ 118.48	*p* < 0.001
	Unhealthy	24.6	36.0	32.3	27.3	38.7	44.7	32.6	45.4		
	mean	0.09	0.13	0.11	0.10	0.15	0.15	0.13	0.15	F*_(7, 989.10)_ = 17.73 *W*_(7, 411.83)_ = 16.42	*p* < 0.001
Well-being (SWLS)	High	64.9	53.2	56.4	55.0	45.0	37.3	67.4	43.9	$\chi_{\left(7\right)}^{2}=$ 155.76	*p* < 0.001
	Low	35.1	46.8	43.6	45.1	55.0	62.8	32.6	56.2		
	mean	0.26	0.33	0.32	0.30	0.36	0.40	0.28	0.34	F*_(7, 949)_ = 30.84 *W*_(7, 409.26)_ = 29.72	*p* < 0.001

*Ps_0_Ph_0_D_0_: Not exposed to psychological abuse, physical abuse and substance abuse distress in childhood. Ps_1_Ph_0_D_0_: Exposed to psychological abuse but not physical abuse and substance abuse distress. Ps_0_Ph_1_D_0_: Exposed to physical abuse but not psychological abuse and substance abuse distress. Ps_0_Ph_0_D_1_: Exposed to substance abuse distress, but not psychological abuse and physical abuse. Ps_1_Ph_1_D_0_: Exposed to both psychological and physical abuse but not substance abuse distress. Ps_1_Ph_0_D_1_: Exposed to both psychological abuse and substance abuse distress but not physical abuse. Ps_0_Ph_1_D_1_: Exposed to both physical abuse and substance abuse distress but not psychological abuse. Ps_1_Ph_1_D_1_: Exposed to psychological abuse, physical abuse, and substance abuse distress*.

a *Social support factors were measured by instrumental and emotional support. Instrumental support: Do you have enough friends who can give you help and support when you need it? (yes, no); Emotional support: Do you have enough friends you can talk confidentially with? (yes, no)*.

b *Behavioral factors were measured by two questions: Do you/did you smoke daily? (yes, now; yes, previously; never); How many units of alcohol (a beer, a glass of wine or a drink) do you usually drink when you drink alcohol? (1–4, 5–6, 7–9, 10 or more)*.

*SCL-10: Mental health status was measured by the Hopkins Symptoms Check List-10 (SCL-10)*.

*EQ-5D: Health was assessed by the EQ-5D generic measure of health-related quality of life*.

*SWLS: Well-being was measured by the satisfaction with life scale (SWLS)*.

c *Test for linear trend p < 0.01*.

### Association between childhood traumatic experiences and mental health, health, and well-being in adulthood

**Figure 1:**
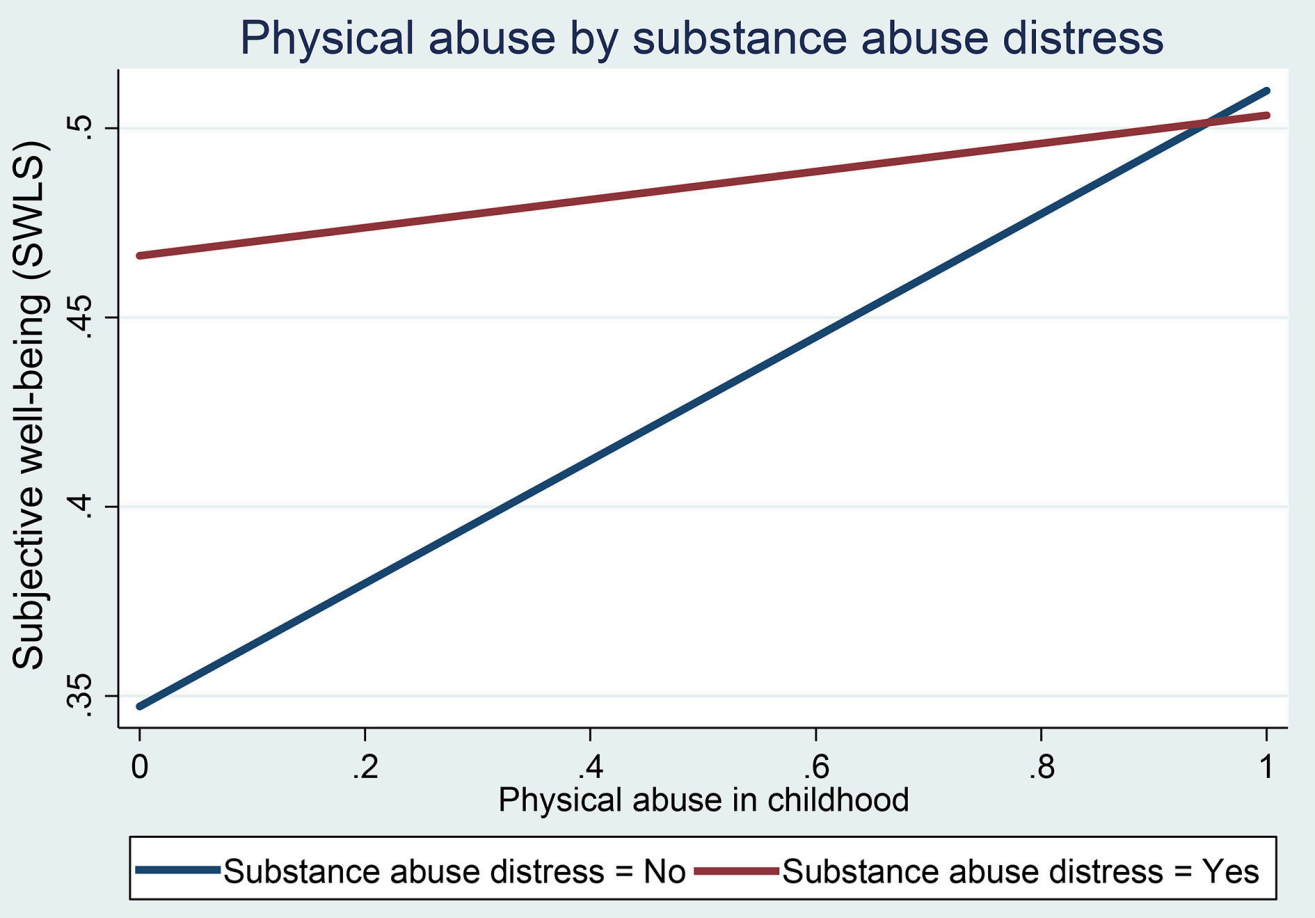
**Effect of physical abuse on well-being (OLS model) by substance abuse distress in childhood**.

The crude association between CTEs and mental health, health, and well-being in adulthood was assessed with linear regression (Figure 1), F^*^ tests, Welch (*W*) tests (Table 7), and cross-tabulation with chi-square tests (Table 7). The F^*^ test is a modification of the standard *F*-test that is more robust to violations of the homogeneity of variance assumption (Wilcox et al., 1986). Similarly, Welch (*W*) test is more robust to violations of homogeneity of variances (Welch, 1947; Wilcox et al., 1986). To assess the linear trend, the *trauma frequency* variable was modeled as a continuous variable in the quantile regression models [using continuous outcomes (scale: 0–1)] (Table 8). Furthermore, we assessed if there was a significant difference between the three CTEs with multiple comparisons (Table 8). The independent influence of CTEs (adjusted for covariates, and mediators) on mental health, health, and well-being was assessed with quantile regression models [using continuous outcomes (scale: 0–1)] (Table 6), and Poisson regression models (using binary outcomes) (**Tables 11**, **12**).

**Table 8:** **Association between childhood traumatic experiences and mental health (SCL-10), health (EQ-5D), and subjective well-being (SWLS) with quantile regression models (*N* = 12,981)**.

		**Imputed dataset with multiple imputation (*n* = 12, 981)**		
		**Mental health (SCL-10)**	**Health (EQ-5D)**	**Well-being (SWLS)**
Childhood traumatic experiences	*n*	β (95% CI)	β (95% CI)	β (95% CI)
Trauma frequency (*reference: not exposed*)^e^, ^i^	10,907	*Ref*	*Ref*	*Ref*
Exposed to any one traumatic experience^e^, ^i^	525	**0.03 (0.01–0.05)**^d^	**0.03 (0.02–0.04)**^d^	**0.06 (0.05–0.06)**^d^
Exposed to any two traumatic experiences^e^, ^i^	1416	**0.03 (0.03–0.11)**^d^	**0.03 (0.02–0.03)**^d^	**0.06 (0.05–0.06)**^d^
Exposed to all three traumatic experiences^e^, ^i^	133	**0.07 (0.03–0.11)**^d^	**0.06 (0.04–0.08)**^d^	**0.06 (0.01–0.10)**^b^
Psychological abuse *vs*. physical abuse (ref)^h^	1431	0.00 (–0.02–0.02)	–0.01 (–0.02–0.00)	0.00 (–0.02–0.02)
Psychological abuse *vs*. substance abuse distress (ref)^g^	1844	**0.03 (0.02–0.05)**^d^	0.01 (–0.01–0.03)	0.00 (–0.02–0.02)
Physical abuse *vs*. substance abuse distress (ref)^f^	1549	–0.00 (–0.02–0.01)	–0.00 (–0.02–0.02)	–0.00 (–0.02–0.02)
Psychological abuse and physical abuse *vs*. substance abuse distress (ref)^e^	1319	**0.04 (0.01–0.06)**^c^	–0.00 (–0.02–0.02)	0.02 (–0.02–0.06)
Psychological abuse and distress *vs*. physical abuse (ref)^e^	906	**0.04 (–0.00–0.09)**^a^	–0.00 (–0.02–0.02)	**0.06 (–0.00–0.11)**^a^
Physical abuse and distress *vs*. psychological abuse (ref)^e^	1201	–0.02 (–0.07–0.04)	–0.01 (–0.06–0.04)	**–0.06 (–0.12–0.01)**^a^
All three traumatic experiences *vs*. psychological abuse and physical abuse only (ref)^e^, ^j^	526	0.03 (–0.02–0.07)	0.02 (–0.01–0.05)	0.00 (–0.05–0.05)
All three traumatic experiences *vs*. psychological abuse and substance abuse distress only (ref)^e^, ^k^	239	0.01 (–0.04–0.07)	0.03 (–0.02–0.08)	0.00 (–0.06–0.06)
All three traumatic experiences *vs*. physical abuse and substance abuse distress only (ref)^e^, ^l^	177	**0.08 (0.01–0.14)**^b^	**0.04 (–0.00–0.09)**^a^	0.06 (–0.01–0.13)

a *P < 0.1*.

b *P < 0.05*.

c *P < 0.01*.

d *P < 0.001*.

e *Adjusted for confounding variables*.

f *Adjusted for confounding variables and psychological abuse*.

g *Adjusted for confounding variables and physical abuse*.

h *Adjusted for confounding variables and substance abuse distress*.

i *Test for linear trend p < 0.001*.

j *Ps_1_Ph_1_D_1_ vs. Ps_1_Ph_1_D_0_ (ref)*.

k *Ps_1_Ph_1_D_1_ vs. Ps_1_Ph_0_D_1_ (ref)*.

l *Ps_1_Ph_1_D_1_vs. Ps_0_Ph_1_D_1_ (ref)*.

*SCL-10: Mental health status was measured by the Hopkins Symptoms Check List-10 (SCL-10); scale (0–1), where 0 represents perfect mental health, and 1 represents worst mental health. EQ-5D: Health was assessed by the EQ-5D generic measure of health-related quality of life; scale (0–1), where 0 represents perfect health, and 1 represents worst health*.

*SWLS: Well-being was measured by the satisfaction with life scale (SWLS); scale (0–1), where 0 represents highest well-being, and 1 represents lowest well-being*.

*All significant associations are in bold*.

### Association between social support and behavioral factors, and mental health, health, and well-being in adulthood

The crude association between social support and behavioral factors, and mental health, health, and well-being was assessed with cross-tabulation with chi-square tests (using binary outcomes) (Table 9). The independent influence (adjusted for covariates) of social support and behavioral factors on mental health, health, and well-being was assessed with quantile regression models [using continuous outcomes (scale: 0–1)] (Table 6) and Poisson regression models (using binary outcomes) (Table 10).

**Table 9:** **Distribution (%) of mediators by mental health (SCL-10), health (EQ-5D), and subjective well-being (SWLS)**.

		**Mental health (SCL-10)**			**Health (EQ-5D)**			**Well-being (SWLS)**		
		**Unhealthy *n* = 902**	**Healthy *n* = 10, 215**	**Test statistic**	**Unhealthy *n* = 3049**	**Healthy *n* = 8564**	**Test statistic**	**Low *n* = 4040**	**High *n* = 6720**	**Test statistic**
**MEDIATORS**										
Instrumental support^a^	Yes	5.8	94.2	$\chi_{\left(1\right)}^{2}=$ 646.99^**^	23.1	76.9	$\chi_{\left(1\right)}^{2}=$ 389.36^**^	33.7	66.3	$\chi_{\left(1\right)}^{2}=$ 456.70^*^
	No	27.6	72.4		49.8	50.2		67.0	33.0	
Emotional support^a^	Yes	5.9	94.0	$\chi_{\left(1\right)}^{2}=$ 460.67^**^	23.6	76.5	$\chi_{\left(1\right)}^{2}=$ 241.54^**^	33.5	66.5	$\chi_{\left(1\right)}^{2}=$ 411.37^*^
	No	23.1	76.9		43.0	57.0		62.4	37.6	
Daily smoking^b^	Never	6.8	93.2	$\chi_{\left(2\right)}^{2}=$ 57.03^**^	21.9	78.1	$\chi_{\left(2\right)}^{2}=$ 90.31^**^	33.7	66.3	$\chi_{\left(2\right)}^{2}=$ 66.63^*^
	Previously	7.5	92.6		26.8	73.2		37.9	62.1	
	Yes	11.9	88.1		32.6	67.4		44.2	55.8	
Alcohol units^b^, ^c^	1–4	7.2	92.8	$\chi_{\left(3\right)}^{2}=$ 48.01^**^	24.6	75.6	$\chi_{\left(3\right)}^{2}=$ 8.43^*^	36.4	63.6	$\chi_{\left(3\right)}^{2}=$ 28.89^*^
	5–6	9.1	90.9		24.9	75.1		40.9	59.1	
	7–9	15.3	84.7		32.0	68.0		48.0	52.0	
	10 or more	25.4	74.6		32.8	67.2		59.3	40.7	

* *p < 0.05*.

** *p < 0.001*.

a *Social support factors were measured by instrumental and emotional support. Instrumental support: Do you have enough friends who can give you help and support when you need it? (yes, no); Emotional support: Do you have enough friends you can talk confidentially with? (yes, no)*.

b *Behavioral factors were measured by two questions: Do you/did you smoke daily? (yes, now; yes, previously; never); How many units of alcohol (a beer, a glass of wine or a drink) do you usually drink when you drink alcohol? (1–4, 5–6, 7–9, 10 or more)*.

*SCL-10: Mental health status was measured by the Hopkins Symptoms Check List-10 (SCL-10)*.

*EQ-5D: Health was assessed by the EQ-5D generic measure of health-related quality of life*.

*SWLS: Well-being was measured by the satisfaction with life scale (SWLS)*.

c *Test for linear trend p < 0.001*.

**Table 10:** **Association between mediators and mental health (SCL-10), health (EQ-5D), and subjective well-being (SWLS)**.

**Mediators^e^, ^f^**		**Complete-case analysis (excluding missing)**		**Imputed dataset with multiple imputation (*****n*** = 12, 981**)**	
		**Unadjusted**	**Adjusted^j^**	**Unadjusted**	**Adjusted^j^**
		**RR (95% CI)**	**RR (95% CI)**	**RR (95% CI)**	**RR (95% CI)**
**MENTAL HEALTH (SCL-10)**					
Instrumental support^e^	No^g^	**4.76 (4.21–5.39)**^d^	**2.53 (1.96–3.28)**^d^	**4.57 (4.10–5.10)**^d^	**2.49 (2.08–2.99)**^d^
Emotional support^e^	No^g^	**3.86 (3.41–4.39)**^d^	**1.88 (1.46–2.44)**^d^	**3.73 (3.34–4.17)**^d^	**1.87 (1.57–2.24)**^d^
Alcohol use (units)^f^, ^h^, ^k^	5–6^h^	**1.27 (0.99–1.61)**^a^	1.23 (0.93–1.61)	1.13 (0.91–1.41)	**1.25 (1.01–1.56)**^b^
	7–9^h^	**2.12 (1.52–2.96)**^d^	**1.98 (1.41–2.77)**^d^	**1.75 (1.28–2.40)**^d^	**1.92 (1.42–2.58)**^d^
	10 or more^h^	**3.53 (2.27–5.50)**^d^	**3.26 (2.17–4.89)**^d^	**2.92 (1.95–4.37)**^d^	**2.54 (1.69–3.85)**^d^
Daily smoking^f^, ^i^	Previous smoker^i^	1.10 (0.94–1.28)	**1.18 (0.98–1.42)**^a^	1.11 (0.97–1.27)	1.09 (0.96–1.24)
	Current smoker^i^	**1.77 (1.51–2.08)**^d^	**1.63 (1.34–1.99)**^d^	**1.76 (1.53–2.02)**^d^	**1.54 (1.34–1.76)**^d^
**HEALTH (EQ-5D)**					
Instrumental support^e^	No^g^	**2.15 (2.01–2.31)**^d^	**1.46 (1.29–1.64)**^d^	**2.12 (1.99–2.26)**^d^	**1.52 (1.38–1.67)**^d^
Emotional support^e^	No^g^	**1.82 (1.70–1.96)**^d^	**1.39 (1.24–1.57)**^d^	**1.80 (1.69–1.92)**^d^	**1.29 (1.18–1.42)**^d^
Alcohol use (units)^f^, ^h^, ^k^	5–6^h^	1.02 (0.90–1.16)	**1.25 (1.09–1.44)**^d^	0.96 (0.85–1.09)	**1.22 (1.08–1.38)**^c^
	7–9^h^	**1.31 (1.07–1.61)**^c^	**1.72 (1.38–2.14)**^d^	**1.24 (1.02–1.50)**^b^	**1.68 (1.39–2.03)**^d^
	10 or more^h^	1.35 (0.94–1.93)	**1.64 (1.12–2.41)**^c^	1.21 (0.86–1.72)	**1.49 (1.05–2.12)**^b^
Daily smoking^f^, ^i^	Previous smoker^i^	1.10 (0.94–1.28)	**1.24 (1.13–1.35)**^d^	**1.19 (1.11–1.27)**^d^	**1.15 (1.08–1.23)**^d^
	Current smoker^i^	**1.77 (1.51–2.08)**^d^	**1.49 (1.35–1.64)**^d^	**1.44 (1.34–1.56)**^d^	**1.38 (1.28–1.49)**^d^
**WELL-BEING (SWLS)**					
Instrumental support^e^	No^g^	**1.99 (1.89–2.10)**^d^	**1.39 (1.28–1.52)**^d^	**1.92 (1.83–2.01)**^d^	**1.40 (1.30–1.51)**^d^
Emotional support^e^	No^g^	**1.86 (1.77–1.96)**^d^	**1.44 (1.33–1.57)**^d^	**1.83 (1.75–1.92)**^d^	**1.40 (1.30–1.50)**^d^
Alcohol use (units)^f^, ^h^, ^k^	5–6^h^	**1.12 (1.02–1.23)**^b^	**1.12 (1.01–1.23)**^b^	**1.12 (1.03–1.22)**^b^	**1.11 (1.01–1.21)**^b^
	7–9^h^	**1.32 (1.14–1.53)**^d^	**1.33 (1.15–1.55)**^d^	**1.27 (1.09–1.47)**^c^	**1.24 (1.07–1.43)**^c^
	10 or more^h^	**1.63 (1.32–2.02)**^d^	**1.48 (1.17–1.86)**^d^	**1.56 (1.26–1.93)**^d^	**1.43 (1.15–1.76)**^d^
Daily smoking^f^, ^i^	Previous smoker^i^	**1.12 (1.06–1.19)**^d^	**1.08 (1.02–1.15)**^b^	**1.10 (1.05–1.17)**^d^	**1.07 (1.02–1.13)**^c^
	Current smoker^i^	**1.31 (1.23–1.40)**^d^	**1.22 (1.13–1.31)**^d^	**1.29 (1.21–1.37)**^d^	**1.22 (1.14–1.29)**^d^

a *P < 0.1*.

b *P < 0.05*.

c *P < 0.01*.

d *P < 0.001*.

e *Social support factors were measured by instrumental and emotional support. Instrumental support: Do you have enough friends who can give you help and support when you need it? (yes, no); Emotional support: Do you have enough friends you can talk confidentially with? (yes, no)*.

f *Behavioral factors were measured by two questions: Do you/did you smoke daily? (yes, now; yes, previously; never); How many units of alcohol (a beer, a glass of wine or a drink) do you usually drink when you drink alcohol? (1–4, 5–6, 7–9, 10 or more)*.

*SCL-10: Mental health status was measured by the Hopkins Symptoms Check List-10 (SCL-10). EQ-5D: Health was assessed by the EQ-5D generic measure of health-related quality of life. SWLS: Well-being was measured by the satisfaction with life scale (SWLS)*.

g *Reference: Yes*.

h *Reference: 1–4 units*.

i *Reference: Never*.

j *Adjusted for childhood traumatic experiences, confounding variables and other mediators*.

k *Test for linear trend p < 0.001*.

*All significant associations are in bold*.

*The binary outcomes of mental health, health, and well-being were used*.

### Assessing direct and indirect effect (through social support and behavioral factors in adulthood) of childhood traumatic experiences on mental health, health, and well-being

The binary variables of mental health, health, and well-being were used for mediation analyses. The three types of CTEs were tested for pairwise multiplicative interaction between them with logistic and Poisson regression models. In addition, all independent variables were tested for pairwise multiplicative interactions with the CTEs combinations, by logistic and Poisson regression models.

As the outcomes were binary, we used the following model to fit the data, in which y = health or well-being outcome; ps = psychological abuse in childhood; ph = physical abuse in childhood; d = substance abuse distress in childhood; and c = covariates:

$$\begin{array}{llll} \; & log\left\{P\left(Y=1|ps,\;ph,\;d,c\right)\right\}=\beta_{0}\;+\;\beta_{1}ps\;+\;\beta_{2}\;ph & \; & \;\\ \; & +\;\beta_{3}\;d+\;\beta_{4}\;ps\;*\;ph\;+\;\beta_{5}\;ps\;*\;d\;+\;\beta_{6}ph\;*\;d & \; & \;\\ \; & +\;\beta_{7}\;ps\;*\;ph\;*\;d\;+\;\beta_{8}\;c & \; & \; \end{array}$$

A statistically significant multiplicative interaction (*p* < 0.05) was observed between the three types of CTEs (see Figure 1). Therefore, we estimated the effect of seven combinations of these CTEs, compared to no traumatic experience, as Ps_i_Ph_i_D_k_, where *i, j*, and *k* represent the values 0 (not exposed) or 1 (exposed) for the three types of CTEs:

- Ps_0_Ph_0_D_0:_ Not exposed to any of the three CTEs (*n* = 10, 907) (reference category);
- Ps_1_Ph_0_D_0_: Exposed to psychological abuse, but not physical abuse or substance abuse distress (*n* = 525);
- Ps_0_Ph_1_D_0_: Exposed to physical abuse, but not psychological abuse or substance abuse distress (*n* = 230);
- Ps_0_Ph_0_D_1_: Exposed to substance abuse distress, but not psychological abuse or physical abuse (*n* = 643);
- Ps_1_Ph_1_D_0_: Exposed to both psychological and physical abuse, but not substance abuse distress (*n* = 393);
- Ps_1_Ph_0_D_1_: Exposed to both psychological abuse and substance abuse distress, but not physical abuse (*n* = 106);
- Ps_0_Ph_1_D_1_: Exposed to both physical abuse and substance abuse distress, but not psychological abuse (*n* = 44);
- Ps_1_Ph_1_D_1_: Exposed to all three CTEs (*n* = 133).

Each combination of CTEs constituted a separate exposure in the analyses. Unadjusted estimates, and estimates adjusted for potential confounding variables are presented (Tables 11, 12). The estimation strategy for assessing mediation was based on prior theory and the Causal Steps method (Judd and Kenny, 1981; Baron and Kenny, 1986). We used the ‘difference method’ approach (Wright, 1934; Judd and Kenny, 1981; Clogg et al., 1992) to assess mediation. An important assumption of assessing mediation is that there is no exposure-mediator multiplicative interaction (Clogg et al., 1992; Robins and Greenland, 1992; Have et al., 2004; Kaufman et al., 2004; Martinussen, 2009; Sheikh et al., 2014). Other assumptions for assessing mediation with multiple mediators include that there is no multiplicative interaction between mediators, or between mediators and covariates. Moreover, when the outcome is not rare, odds ratios are not suitable for assessing mediation (Pearl, 2012; Sheikh et al., 2014), as the direct effect is overestimated, and the indirect effect is underestimated (Jiang and VanderWeele, 2015) due to the property of non-collapsibility (Miettinen and Cook, 1981; Greenland, 1987; Greenland et al., 1999; Pang et al., 2013). Therefore, Poisson regression analysis (RR and 95% CIs) with robust error variance (Barros and Hirakata, 2003; Zou, 2004) was used to estimate the total and direct effect of CTEs on mental health, health, and well-being. Mediators were included in the models to assess the indirect effect in the form of *proportion of mediated effect (% attenuation)* (Susser, 1973; Szklo and Nieto, 2000). We calculated 95% CIs for indirect effects using a bias-corrected accelerated bootstrap method (Carpenter and Bithell, 2000) with 2000 re-samplings. The *% attenuation* (indirect effect) was not estimated when the RR (of total effect or direct effect) was less than 1.00, or when there was no reduction in the RR_Total Effect_ after including the mediators in the model (Tables 11, 12). Mediation was assessed in both the complete-case dataset (excluding missing) (Table 11), and the imputed dataset with MI (Table 12).

**Table 11:** **Effect of exposure to childhood traumatic experiences on mental health (SCL-10), health (EQ-5D), and subjective well-being (SWLS)**.

	**Crude effects**	**Total effects**	**Direct effects**	**Proportion mediated (Indirect effects)**
	**Unadjusted**	**Adjusted for confounding variables^c^**	**Adjusted for confounding variables^c^**and mediators**^a^, ^b^**	
**Childhood traumatic experiences (CTEs)**	**RR (95% CI)**	**RR (95% CI)**	**RR (95% CI)**	**%attentuation**^d^ **(95% CI)**
**MENTAL HEALTH (SCL-10)** *n* = 8547					
No trauma (Ps_0_Ph_0_D_0_)	1.00	1.00	1.00	Ref
Psychological abuse only (Ps_1_Ph_0_D_0_)	**2.29(1.84–2.87)**	**2.19(1.67–2.87)**	**1.92(1.49–2.47)**	**22.88(2.96–40.26)**
Physical abuse only (Ps_0_Ph_1_D_0_)	1.11(0.69–1.80)	1.10(0.61–1.97)	1.08(0.61–1.92)	17.82(–42.84–4455.85)
Substance abuse distress only (Ps_0_Ph_0_D_1_)	**1.39(1.07–1–82)**	1.02(0.73–1.43)	0.93(0.68–1.29)	–
Psychological and physical abuse (Ps_1_Ph_1_D_0_)	**2.58(2.03–3.28)**	**2.69(2.03–3.58)**	**1.99(1.52–2.60)**	**41.60(24.67–56.27)**
Psychological abuse and substance abuse distress (Ps_1_Ph_0_D_1_)	**3.27(2.23–4.80)**	**2.14(1.23–3.73)**	1.51(0.89–2.56)	**54.97(1.38–153.99)**
Physical abuse and substance abuse distress (Ps_0_Ph_1_D_1_)	1.91(0.84–4.33)	2.22(0.88–5.57)	2.37(0.95–5.95)	–
Psychological abuse, physical abuse and substance abuse distress (Ps_1_Ph_1_D_1_)	**3.85(2.81–5.27)**	**3.72(2.67–5.20)**	**3.10(2.20–4.35)**	23.01(–1.08–42.94)
**HEALTH (EQ-5D)** *n* = 9312					
No trauma (Ps_0_Ph_0_D_0_)	1.00	1.00	1.00	Ref
Psychological abuse only (Ps_1_Ph_0_D_0_)	**1.47(1.30–1.66)**	**1.57(1.37–1.82)**	**1.51(1.21–1.73)**	**8.50(0.40–18.11)**
Physical abuse only (Ps_0_Ph_1_D_0_)	**1.32(1.08–1.60)**	**1.45(1.14–1.76)**	**1.38(1.10–1.68)**	12.49(–1.32–34.75)
Substance abuse distress only (Ps_0_Ph_0_D_1_)	1.11(0.97–1.27)	1.11(0.93–1.27)	1.07(0.93–1.21)	36.11(–47.57–293.16)
Psychological and physical abuse (Ps_1_Ph_1_D_0_)	**1.58(1.38–1.80)**	**1.78(1.50–2.03)**	**1.54(1.32–1.77)**	**24.41(15.90–36.83)**
Psychological abuse and substance abuse distress (Ps_1_Ph_0_D_1_)	**1.82(1.46–2.26)**	**1.61(1.14–2.12)**	1.44(0.98–1.89)	**24.14(2.88–91.34)**
Physical abuse and substance abuse distress (Ps_0_Ph_1_D_1_)	1.33(0.86–2.04)	1.69(0.91–2.61)	1.63(0.88–2.50)	7.13(–24.50–74.68)
Psychological abuse, physical abuse and substance abuse distress (Ps_1_Ph_1_D_1_)	**1.85(1.53–2.24)**	**2.12(1.66–2.55)**	**1.96(1.54–2.37)**	**10.56(1.61–20.92)**
**WELL-BEING (SWLS)** *n* = 8965					
No trauma (Ps_0_Ph_0_D_0_)	1.00	1.00	1.00	Ref
Psychological abuse only (Ps_1_Ph_0_D_0_)	**1.33(1.21–1.47)**	**1.29(1.15–1.43)**	**1.25(1.13–1.39)**	**13.55 (2.32–30.25)**
Physical abuse only (Ps_0_Ph_1_D_0_)	**1.24(1.06–1.45)**	1.16(0.97–1.37**)**	1.12(0.93–1.32)	25.02(–13.72–217.20)
Substance abuse distress only (Ps_0_Ph_0_D_1_)	**1.28(1.17–1.41)**	**1.24(1.11–1.37)**	**1.19(1.07–1.31)**	**17.60(6.33–34.57)**
Psychological and physical abuse (Ps_1_Ph_1_D_0_)	**1.57(1.43–1.73)**	**1.48(1.32–1.65)**	**1.31(1.19–1.46)**	**31.24(20.08–45.73)**
Psychological abuse and substance abuse distress (Ps_1_Ph_0_D_1_)	**1.79(1.54–2.08)**	**1.62(1.31–1.92)**	**1.46(1.17–1.73)**	**22.36(5.64–43.85)**
Physical abuse and substance abuse distress (Ps_0_Ph_1_D_1_)	0.93(0.60–1.43)	0.87(0.47–1.34)	0.84(0.47–1.24)	–
Psychological abuse, physical abuse and substance abuse distress (Ps_1_Ph_1_D_1_)	**1.60(1.37–1.87)**	**1.46(1.21–1.75)**	**1.37(1.15–1.61)**	**16.59(1.41–39.56)**

*The binary outcomes of mental health, health, and well-being were used for mediation analysis*.

*Ps_0_Ph_0_D_0_: Not exposed to psychological abuse, physical abuse and substance abuse distress in childhood. Ps_1_Ph_0_D_0_: Exposed to psychological abuse but not physical abuse and substance abuse distress. Ps_0_Ph_1_D_0_: Exposed to physical abuse but not psychological abuse and substance abuse distress. Ps_0_Ph_0_D_1_: Exposed to substance abuse distress, but not psychological abuse and physical abuse. Ps_1_Ph_1_D_0_: Exposed to both psychological and physical abuse but not substance abuse distress. Ps_1_Ph_0_D_1_: Exposed to both psychological abuse and substance abuse distress but not physical abuse. Ps_0_Ph_1_D_1_: Exposed to both physical abuse and substance abuse distress but not psychological abuse. Ps_1_Ph_1_D_1_: Exposed to psychological abuse, physical abuse, and substance abuse distress*.

a *Social support factors were measured by instrumental and emotional support. Instrumental support: Do you have enough friends who can give you help and support when you need it? (yes, no); Emotional support: Do you have enough friends you can talk confidentially with? (yes, no)*.

b *Behavioral factors were measured by two questions: Do you/did you smoke daily? (yes, now; yes, previously; never); How many units of alcohol (a beer, a glass of wine or a drink) do you usually drink when you drink alcohol? (1–4, 5–6, 7–9, 10 or more)*.

c *Confounding variables were age, gender, father's education, mother's education and childhood financial conditions*.

d The percentages show the reduction in relative risk (RR) in model adjusted for mediators, compared to model adjusted only for confounding variables. For instance, the reduction in the RR for mental health for the Ps_1_Ph_0_D_0_group when including mediators to the first model, is [(2.19 – 1.92) / (2.19 – 1.00)] * 100 = 22.88%.

*All significant associations (p < 0.05) are in bold*.

*SCL-10: Mental health status was measured by the Hopkins Symptoms Check List-10 (SCL-10)*.

*EQ-5D: Health was assessed by the EQ-5D generic measure of health-related quality of life*.

*SWLS: Well-being was measured by the satisfaction with life scale (SWLS)*.

**Table 12:** **Effect of childhood traumatic experiences on mental health (SCL-10), health (EQ-5D), and subjective well-being (SWLS) in imputed dataset with multiple imputation (*N* = 12,981)**.

	**Crude effects**	**Total effects**	**Direct effects**	**Indirect effects**
	**Unadjusted**	**Adjusted for confounding variables^c^**	**Adjusted for confounding variables^c^ and mediators^a^, ^b^**	**Proportion mediated^±^**
	**RR (95% CI)**	**RR (95% CI)**	**RR (95% CI)**	**%attentuation (95% CI)**
**MENTAL HEALTH (SCL-10)**				
No trauma (Ps_0_Ph_0_D_0_)	1.00 (ref)	1.00 (ref)	1.00 (ref)	Ref
Psychological abuse only (Ps_1_Ph_0_D_0_)	**2.04 (1.66–2.51)**	**1.94 (1.57–2.17)**	**1.62 (1.47–1.90)**	**25.63 (17.42–39.59)**
Physical abuse only (Ps_0_Ph_1_D_0_)	1.06 (0.69–1.64)	1.12 (0.68–1.65)	1.01 (0.59–1.45)	90.96 (–36.23–155.56)
Substance abuse distress only (Ps_0_Ph_0_D_1_)	**1.38 (1.09–1–73)**	**1.22 (1.07–1.38)**	1.11 (0.92–1.24)	**46.37 (17.15–2343–.24)**
Psychological and physical abuse (Ps_1_Ph_1_D_0_)	**2.41 (1.95–2.98)**	**2.30 (1.96–2.63)**	**1.73 (1.55–2.03)**	**34.44 (26.60–37.47)**
Psychological abuse and substance abuse distress (Ps_1_Ph_0_D_1_)	**2.93 (2.06–4.16)**	**2.25 (1.92–2.94)**	**1.80 (1.55–2.28)**	**27.49 (19.17–36.55)**
Physical abuse and substance abuse distress (Ps_0_Ph_1_D_1_)	1.41 (0.61–3.22)	1.32 (0.48–1.74)	1.28 (0.52–1.86)	10.83 (–40.78–86.25)
Psychological abuse, physical abuse and substance abuse distress (Ps_1_Ph_1_D_1_)	**3.32 (2.49–4.44)**	**2.75 (2.19–3.10)**	**2.36 (1.89–2.82)**	**15.04 (8.94–27.16)**
**HEALTH (EQ-5D)**				
No trauma (Ps_0_Ph_0_D_0_)	1.00 (ref)	1.00 (ref)	1.00 (ref)	Ref
Psychological abuse only (Ps_1_Ph_0_D_0_)	**1.40 (1.25–1.58)**	**1.51 (1.44–1.53)**	**1.43 (1.42–1.43)**	**12.83 (9.10–19.37)**
Physical abuse only (Ps_0_Ph_1_D_0_)	**1.30 (1.08–1.56)**	**1.42 (1.33–1.60)**	**1.34 (1.21–1.42)**	**16.69 (13.35–33.10)**
Substance abuse distress only (Ps_0_Ph_0_D_1_)	1.06 (0.93–1.21)	1.08 (0.95–1.14)	1.04 (0.93–1.10)	43.65 (–62.06–78.78)
Psychological and physical abuse (Ps_1_Ph_1_D_0_)	**1.50 (1.32–1.71)**	**1.63 (1.47–1.79)**	**1.45 (1.33–1.64)**	**24.27 (18.59–31.55)**
Psychological abuse and substance abuse distress (Ps_1_Ph_0_D_1_)	**1.74 (1.41–2.16)**	**1.80 (1.48–2.18)**	**1.65 (1.37–1.93)**	**14.06 (5.99–26.57)**
Physical abuse and substance abuse distress (Ps_0_Ph_1_D_1_)	1.23 (0.80–1.90)	**1.41 (1.12–1.86)**	**1.36 (1.10–1.82)**	11.20 (–5.80–41.01)
Psychological abuse, physical abuse and substance abuse distress (Ps_1_Ph_1_D_1_)	**1.77 (1.46–2.13)**	**1.89 (1.47–1.99)**	**1.76 (1.43–1.86)**	**11.53 (4.06–19.01)**
**WELL-BEING (SWLS)**				
No trauma (Ps_0_Ph_0_D_0_)	1.00 (ref)	1.00 (ref)	1.00 (ref)	Ref
Psychological abuse only (Ps_1_Ph_0_D_0_)	**1.34 (1.22–1.47)**	**1.27 (1.20–1.35)**	**1.21 (1.13–1.27)**	**20.24 (15.92–30.55)**
Physical abuse only (Ps_0_Ph_1_D_0_)	1.14 (0.98–1.32)	**1.18 (1.12–1.28)**	**1.13 (1.05–1.21)**	**29.34 (9.62–53.57)**
Substance abuse distress only (Ps_0_Ph_0_D_1_)	**1.20 (1.09–1.32)**	**1.20 (1.13–1.26)**	**1.16 (1.13–1.22)**	**18.09 (10.65–27.64)**
Psychological and physical abuse (Ps_1_Ph_1_D_0_)	**1.48 (1.34–1.64)**	**1.46 (1.37–1.51)**	**1.30 (1.25–1.37)**	**29.32 (28.14–33.05)**
Psychological abuse and substance abuse distress (Ps_1_Ph_0_D_1_)	**1.72 (1.47–2.01)**	**1.62 (1.42–2.00)**	**1.49 (1.29–1.77)**	**17.18 (8.19–27.91)**
Physical abuse and substance abuse distress (Ps_0_Ph_1_D_1_)	0.87 (0.51–1.48)	0.91 (0.72–1.19)	0.89 (0.67–1.14)	–
Psychological abuse, physical abuse and substance abuse distress (Ps_1_Ph_1_D_1_)	**1.44 (1.21–1.71)**	**1.42 (1.29–1.52)**	**1.33 (1.21–1.44)**	**18.18 (11.33–30.10)**

*The binary outcomes of mental health, health, and well-being were used for mediation analysis*.

*Ps_*0*_Ph_*0*_D_*0*_: Not exposed to psychological abuse, physical abuse and substance abuse distress in childhood. Ps_*1*_Ph_*0*_D_*0*_: Exposed to psychological abuse but not physical abuse and substance abuse distress. Ps_*0*_Ph_*1*_D_*0*_: Exposed to physical abuse but not psychological abuse and substance abuse distress. Ps_*0*_Ph_*0*_D_*1*_: Exposed to substance abuse distress, but not psychological abuse and physical abuse. Ps_*1*_Ph_*1*_D_*0*_: Exposed to both psychological and physical abuse but not substance abuse distress. Ps_*1*_Ph_*0*_D_*1*_: Exposed to both psychological abuse and substance abuse distress but not physical abuse. Ps_*0*_Ph_*1*_D_*1*_: Exposed to both physical abuse and substance abuse distress but not psychological abuse. Ps_*1*_Ph_*1*_D_*1*_: Exposed to psychological abuse, physical abuse, and substance abuse distress*.

a *Social support factors were measured by instrumental and emotional support. Instrumental support: Do you have enough friends who can give you help and support when you need it? (yes, no); Emotional support: Do you have enough friends you can talk confidentially with? (yes, no)*.

b *Behavioral factors were measured by two questions: Do you/did you smoke daily? (yes, now; yes, previously; never); How many units of alcohol (a beer, a glass of wine or a drink) do you usually drink when you drink alcohol? (1–4, 5–6, 7–9, 10 or more)*.

c *Confounding variables were age, gender, father's education, mother's education and childhood financial conditions*.

± The percentages show the reduction in relative risk (RR) in model adjusted for mediators, compared to model adjusted only for confounding variables. For instance, the reduction in the RR for mental health for the Ps_*1*_Ph_*0*_D_*0*_ group when including mediators to the first model, is [(1.94 – 1.62) / (1.94 – 1.00)] * 100 = 25.63%.

*SCL-10: Mental health status was measured by the Hopkins Symptoms Check List-10 (SCL-10)*.

*EQ-5D: Health was assessed by the EQ-5D generic measure of health-related quality of life*.

*SWLS: Well-being was measured by the satisfaction with life scale (SWLS)*.

*All significant associations (p < 0.01) are in bold*.

## Results

Tables 1, 2 presents the general characteristics of the study sample. The numbers for mother's education, father's education, and childhood financial conditions do not add up to 12,981 due to missing values. Proportions (%) of respondents in the imputed dataset are also presented (Tables 1, 2). The numbers and proportions (%) of the combinations of childhood traumatic experiences show that there is a considerable overlap between the three exposures (Table 1).

The majority (77.9%) of the respondents were between 40 and 69 years of age, reported primary and secondary school or similar as mother's (78.7%) and father's education (64.2%), and reported having good financial conditions in childhood (66.6%) (Table 1). The distributions of variables were similar in the complete-case dataset (excluding those with missing values) and the imputed dataset with MI (Tables 1, 2).

### Relative contribution of socio-demographic factors, childhood socioeconomic status, childhood traumatic experiences to social support, and behavioral factors in adulthood

Table 3 presents the average marginal contribution of all explanatory variables to social support and behavioral factors. The Shapley decomposition shows that among all the variables considered, childhood financial conditions was most important to instrumental support (40.50%), gender was most important to emotional support (35.73%), age was most important to alcohol use (53.19%), while father's education was most important to smoking (32.52%).

However, when the socio-demographic variables (gender, age) and CSES (mother's education, father's education and childhood financial conditions) were considered together, CSES explained most variation in instrumental support (43.23%), and smoking (67.36%), while socio-demographic variables explained most variation in emotional support (43.99%) and alcohol use (94.37%) (Table 3).

### Relative contribution of socio-demographic factors, childhood socioeconomic status, childhood traumatic experiences, social support, and behavioral factors to mental health, health, and well-being

Table 4 presents the average marginal contribution of all explanatory variables used in this study. The Shapley decomposition shows that among all the variables considered, instrumental support (24.16%) explained most of the variation in mental health, while gender (21.32%) explained most of the variation in health, and emotional support (23.34%) explained most of the variation in well-being.

Among all the indicators of childhood adversities, psychological abuse (12.13%) was most important for mental health in adulthood, followed by childhood financial conditions (6.02%), physical abuse (5.30%), substance abuse distress (2.73%), mother's education (0.93%), and father's education (0.61%). While for health in adulthood, childhood financial conditions (10.60%) was most important, followed by psychological abuse (7.01%), mother's education (4.44%), physical abuse (4.19%), father's education (2.68%), and substance abuse distress (1.72%). Furthermore, for well-being, childhood financial conditions (20.60%) was most important, followed by psychological abuse (9.09%), substance abuse distress (4.72%), physical abuse (3.63%), mother's education (2.27%), and father's education (1.30%). However, when the CTEs were considered together, they were relatively more important for mental health than the three indicators of CSES (20.16% for CTEs *vs*. 7.56% for CSES). The three indicators of CSES were relatively more important for health (17.72% *vs*. 12.92%) and well-being (24.17% *vs*. 17.44%) than CTEs (Table 4).

Similarly, if both the social support factors are considered together, they explain most of the variation in mental health (44.78%), health (22.89%) and well-being (43.29%). Among the indicators of social support and behavioral factors, instrumental support was most important for mental health (24.16%) and health (12.02%), followed by emotional support and current smoking. While for well-being, emotional support (23.34%) was most important, followed by instrumental support (19.95%) and current smoking (6.88%).

### Relative contribution of socio-demographic factors, childhood socioeconomic status, childhood traumatic experiences, social support, behavioral factors and mental health to health, and well-being

Table 5 shows that when the relative importance of mental health is taken into consideration for health, it explained most variation in health (75.61%), followed by socio-demographic factors (8.45%), social support factors (5.35%), CSES (4.06%), CTEs (3.31%), and behavioral factors (3.22%). However, for well-being, mental health explained most variation (51.33%), followed by health (22.89%), social support factors (15.25%), CSES (5.07%), CTEs (3.29%), behavioral factors (1.63%) and socio-demographic factors (0.54%).

### Independent influence of each explanatory variable on mental health, health, and well-being

All the independent variables used in this study were included as predictors in the quantile regression model (adjusted for each other) (Table 6). Compared to males, females had lower mental health, health, and well-being (*p* < 0.01) (Table 6). Increased age was associated with lower health (β = 0.001, 95% CI: 0.001–0.001, *p* < 0.001), but higher well-being (β = –0.001, *p* < 0.01) (Table 6). Lower mother's education was associated with lower health and well-being (*p* < 0.001); however, higher father's education was associated with lower well-being (β = 0.005, 95% CI: 0.001–0.009) (Table 6). Having lower financial conditions in childhood was associated with lower mental health, health, and well-being in adulthood (*p* < 0.001) (Table 6). Having no instrumental support or emotional support was associated with lower mental health, health, and well-being (*p* < 0.001). Similarly, higher alcohol use and smoking was associated with lower mental health, health, and well-being in adulthood (*p* < 0.01) (Table 6).

Exposure to psychological abuse (Ps_1_Ph_0_D_0_), psychological and physical abuse (Ps_1_Ph_1_D_0_), psychological abuse and substance abuse distress (Ps_1_Ph_0_D_1_), and exposure to all three CTEs (Ps_1_Ph_1_D_1_) was significantly associated with lower mental health, health, and well-being (Table 6). However, exposure to physical abuse (Ps_0_Ph_1_D_0_), and substance abuse distress (Ps_0_Ph_0_D_1_) was significantly associated with lower mental health and well-being, but not lower health (*p* > 0.1) (Table 6).

### Associations between childhood traumatic experiences, and social support and behavioral factors

All mediators were significantly associated with CTEs (Table 7). The crude associations were mostly in the predicted direction, i.e., respondents with any CTE tend to be current or former smokers, were likely to have no friends to get support from and talk to, and drank five or more units of alcohol whenever they drank (Table 7). The crude associations indicate that for some combinations of CTEs, respondents were more likely to have friends to talk to, and be never smokers (Table 7). However, when the models were adjusted for all covariates, the direction of the association changed to what was expected, i.e., exposure to CTEs were associated with disadvantageous mediator values (data not shown). The test for linear trend (*p* < 0.01) showed that CTEs were associated with higher alcohol use (Table 7). In addition, the crude association between CTEs and the selected mediators was assessed with correspondence analysis (Greenacre, 2007, 2010). See Figure S3 in Online Supplementary Material.

### Association between childhood traumatic experiences and mental health, health, and well-being with quantile regression model

Table 8 presents the association between different combinations of CTEs and mental health, health, and well-being. The test of linear trend (*p* < 0.001) shows that *trauma frequency* was associated with lower mental health, health, and well-being. However, the estimates showed that the association may not be linear. Compared to those with no CTEs, exposure to any *one* or *two* types of CTEs led to similar association for both mental health (β = 0.03, *p* < 0.001), and health (β = 0.03, *p* < 0.001). Similarly, the association with well-being (β = 0.06, *p* < 0.05) remained the same for being exposed to any *one*, any *two*, or all three CTEs (Table 8).

Compared to those exposed to substance abuse distress, exposure to psychological abuse was associated with lower mental health (β = 0.03, 95% CI: 0.02–0.05, *p* < 0.001). Similarly, compared to those exposed to substance abuse distress, exposure to both psychological abuse and physical abuse was associated with lower mental health (β = 0.04, 95% CI: 0.01–0.06, *p* < 0.01).

Compared to those exposed to physical abuse, exposure to both psychological abuse and substance abuse distress was associated with lower mental health (β = 0.03, *p* < 0.1), and well-being (β = 0.06, *p* < 0.1). Similarly, compared to those exposed to both physical abuse and substance abuse distress, exposure to psychological abuse was associated with lower well-being (β = 0.06, *p* < 0.1). Moreover, compared to those exposed to both physical abuse, and substance abuse distress, exposure to all the three CTEs was associated with lower mental health (β = 0.08, 95% CI: 0.01–0.14), and health (β = 0.04, *p* < 0.1) (Table 8).

### Associations between social support, behavioral factors, and mental health, health, and well-being

Table 9 shows the distribution of mediators by mental health, health, and well-being. The crude distributions between mediators and health and well-being showed that all mediators were significantly associated with mental health, health, and well-being in adulthood (Table 9). The crude associations were in the predicted direction, i.e., those who were current or former smokers, had no friends to get support from and talk to, and drank five or more units of alcohol whenever they drank, tend to be unhealthy and have a low level of well-being (Table 9). In addition, the crude association between mediators, and mental health, health, and well-being (using alternative cut-offs for health and well-being) was assessed with correspondence analysis (Greenacre, 2007, 2010). See Figure S4 in Online Supplementary Material.

Table 10 shows the association between mediators and mental health, health and well-being. Both the complete-case analysis (excluding missing), and the imputed dataset analysis are presented. Analyses conducted on the imputed dataset showed that the estimates for mental health, health, and well-being were mostly similar to those in the complete-case dataset. Having no instrumental support, no emotional support, higher alcohol use, and being a daily smoker was associated with an increased risk of being mentally unhealthy, unhealthy, and a low level of well-being (Table 10).

Among the two indicators of social support, having *no* instrumental support led to a higher risk for being mentally unhealthy (RR = 2.49 for instrumental support *vs*. RR = 1.87 for emotional support) and unhealthy (RR = 1.52 for instrumental support *vs*. RR = 1.29 for emotional support), while having *no* instrumental support or emotional support led to a similar increased risk for having a low level of well-being (RR = 1.40) (Table 10). The test for linear trend (*p* < 0.001) showed that increased alcohol use was associated with being unhealthy and having a low level of well-being (Table 10).

### Total effects: the association between childhood traumatic experiences and mental health, health, and well-being in adulthood

Statistically significant multiplicative interactions (*p* < 0.05) were observed between the three CTEs in childhood (data not shown). Figure 1 shows the interaction between physical abuse and substance abuse distress on subjective well-being scale (from unadjusted OLS model). The influence of physical abuse on well-being changed depending on exposure to substance abuse distress (Figure 1).

Those exposed to CTEs tend to be unhealthy and have a low level of well-being (Tables 6–8). All potential confounding variables and mediators were assessed for multiplicative interactions with the CTE combinations. However, there was no evidence of interaction beyond what would be expected by chance alone (data not shown) for any of our outcomes, and the cross-product terms were not included in the final models.

Tables 11, 12 presents the estimates for the risk of being mentally unhealthy, unhealthy, and having a low level of well-being with exposure to CTEs (reference: not exposed to any of the three CTEs). Four estimates are presented: crude (unadjusted), adjusted for confounding variables (total effects), adjusted for confounding variables and mediators (direct effects) and indirect effects in the form of proportion mediated (% attenuation). The unadjusted (crude) associations show that being exposed to most combinations of CTEs significantly (*p* < 0.05) increased the risk of being mentally unhealthy, unhealthy and having a low level of well-being (Table 11).

Table 12 presents the analyses on imputed dataset with MI. After controlling for confounding variables, there was a significant, increased risk of being unhealthy and of having a low level of well-being for most combinations of CTEs (Table 12).

### Direct effects: the association between childhood traumatic experiences and mental health, health, and well-being in adulthood, adjusted for social support and behavioral factors

Tables 11, 12 presents the direct effects (adjusted for confounding variables and mediators). The direct effects show that exposure to psychological abuse only (Ps_1_Ph_0_D_0_), exposure to both psychological and physical abuse (Ps_1_Ph_1_D_0_), and exposure to all the three CTEs (Ps_1_Ph_1_D_1_) in childhood significantly (*p* < 0.05) increased the risk of being mentally unhealthy (Table 11). However, for health and well-being, exposure to most combinations of CTEs significantly (*p* < 0.05) increased the risk of being unhealthy, and having a low level of well-being (Table 11).

The results from the imputed dataset showed that exposure to most combinations of CTEs significantly (*p* < 0.01) increased the risk of being unhealthy, and having a low level of well-being (Table 12). Generally, the risk for being mentally unhealthy was greater than being unhealthy (Table 12). Similarly, the risk for being unhealthy was greater than having a low level of well-being (Table 12). Exposure to psychologically abuse was associated with a higher risk for being mentally unhealthy, unhealthy, and having a low level of well-being. Those exposed to all three types of CTEs (Ps_1_Ph_1_D_1_) had a more than two-fold increased risk of being mentally unhealthy (*RR*_Direct Effect_ = 2.36, 95% CI: 1.89–2.82), a 76% increased risk of being unhealthy (*RR*_Direct Effect_ = 1.76, 95% CI: 1.43–1.86), and a 33%.increased risk of having a low level of well-being (*RR*_Direct Effect_ = 1.33, 95% CI: 1.21–1.44) (Table 12).

Analysis conducted on the imputed dataset (Table 12) show that the estimates for direct effects (and 95% CIs) for health and well-being were nearly the same as those in the complete-case dataset presented in Table 11. However, the estimates for mental health had a slightly different magnitude. After controlling for confounding variables and mediators, the complete-case analyses might even suggest a protective effect of being exposed to substance abuse distress only (*RR*_Direct Effect_ = 0.93, 95% CI: 0.68–1.29) (Table 11), while in the imputed dataset the *RR*_Direct Effect_ estimate was 1.11 (95% CI: 0.92–1.24) (Table 12), which is more plausible.

In most of the models, there was a statistically significant association between CTEs and mental health, health, and well-being after adjusting for mediators. Furthermore, some of the models that were not statistically significant (*p* ≥ 0.05) in the complete-case dataset (Table 11) were statistically significant (*p* < 0.01) in the imputed dataset (Table 12), and were in the same direction.

### Indirect effects: the proportion of mediated effect (% attenuation) by social support and behavioral factors

Tables 11, 12 presents the indirect effects (proportion mediated). The role of mediators was explored by including them in the model adjusted for confounding variables (Tables 11, 12). After adjusting for mediators, the estimates for both psychological abuse and physical abuse (Ps_1_Ph_1_D_0_) were attenuated by 24–41%, while the estimates for both psychological abuse and substance abuse (Ps_1_Ph_0_D_1_) were attenuated by 22–54% (Table 11).

Results from the imputed dataset (Table 12) showed that some of the indirect effects may be underestimated in the complete-case analyses (Table 11). After adjusting for mediators, the estimates for psychological abuse only (Ps_1_Ph_0_D_0_) and health and well-being were attenuated by 12–25%; while the associations with exposure to all the three CTEs were attenuated by 11–18% (Table 12). The indirect effects for health and well-being when using the alternative cut offs (See Tables S7, S8 in Online Supplementary Material) showed that they were very similar to the indirect effects presented in Tables 11, 12. This shows that the indirect effects are robust to how the cut offs are made for health and well-being outcomes.

In summary, the results showed a direct effect of psychological abuse, physical abuse, and substance abuse distress in childhood on mental health, health, and well-being in adulthood, and that some of this affect was mediated through social support and behavioral factors in adulthood.

## Discussion and conclusion

Among all the variables considered, CSES explained most variation in instrumental support and smoking, while socio-demographic variables explained most variation in emotional support and alcohol use. Instrumental support explained most of the variation in mental health, while gender explained most of the variation in health, and emotional support explained most of the variation in well-being. CTEs were relatively more important to mental health than CSES. However, CSES were relatively more important to health and well-being than CTEs. Social support factors were relatively more important to mental health, health, and well-being, as compared to behavioral factors. Moreover, when mental health was included, it explained most variation in both health, and well-being.

Exposure to psychological abuse, both psychological and physical abuse, and both psychological abuse and substance abuse distress was associated with lower mental health, health, and well-being. Similarly, exposure to all the three CTEs was also associated with lower mental health, health, and well-being. Exposure to CTEs was associated with having no social support, being a smoker, and a higher alcohol consumption. Consequently, social support and behavioral factors were associated with lower mental health, health, and well-being.

Consistent with previous studies (Dias et al., 2014), significant multiplicative interactions were observed between the three CTEs. We observed a direct effect of CTEs on adult health and well-being after controlling for selected social support and behavioral factors, but some of this effect was mediated by these factors. These findings support the “chain of risk” model proposed in life course epidemiology. A naïve interpretation would be that a substantial effect of CTEs on adult health and well-being can be reduced by programs/interventions aimed at improving conditions for a social life, and reducing cigarette and alcohol consumption. This paper shows that structural conditions such as CTEs act partly through agency-driven social support and behavioral patterns in adulthood to determine health and well-being. Thus, the standard approach for targeting these individuals to change their behavior will fail as long as these structural conditions remain, as these conditions will pave the way for new mediators to emerge. Assuming that people have a choice to quit smoking, drink less alcohol, and keep an active social life for better health and well-being may not be correct. We have only looked at three types of CTEs, and there can be many structural conditions (Korbin et al., 1998; Coulton et al., 1999) deeply rooted in any society which prevent individuals from making choices freely.

Other models proposed in the life course epidemiology theory include the “sensitive period model,” and the “critical period model” (Ben-Shlomo and Kuh, 2002). The sensitive period model suggests that exposure during a sensitive period may have stronger effects on health, whereas the critical period model suggests that the effect of exposure is only influential if it occurs at a certain “critical” period of life. Our findings are not related to either of these models, since we did not assess the relative importance of similar traumatic experiences from different periods of life.

Contrary to the findings by Lamu and Olsen (2016) health seemed to be more important to subjective well-being, as compared to social support factors. The results were consistent when we used the EQ visual analog scale of health (EQ VAS), and preference-based utility scores of EQ-5D (data not shown). A notable feature of the Shapley decomposition is that it is a function of the group of predictors considered (Hoyos and Narayan, 2011). This is a result of the fact that the Shapley decomposition is sensitive only to variation between predictors, which would naturally vary with the number of predictors considered. Therefore, the relative contribution of the different life circumstances presented here may vary from that presented in other studies. Furthermore, consistent with previous findings (Mäkinen et al., 2006; Sheikh et al., 2014), mother's/father's education is relatively less important for health and well-being, as compared to the subjective assessment of childhood financial conditions. No previous study was found that assessed the relative contribution of CSES, CTEs, and behavioral factors for mental health, health, and well-being.

The results of this study show that the association between CTEs and health and well-being in adulthood varies substantially depending on the type of traumatic experience. This clearly indicates that the unique effect of each type of CTEs is lost by using the sum score of trauma frequency. In line with previous studies, we showed that CTEs are associated with mental health, health, and well-being in adulthood independent of risk factors in adulthood. Consistent with previous studies (Ney, 1987; Martin et al., 2006; Norman et al., 2012; Dias et al., 2014; Spinazzola et al., 2014; Auslander et al., 2015; Friborg et al., 2015), psychological abuse in childhood was associated with a higher risk for being mentally unhealthy and unhealthy, as compared to physical abuse.

Most previous studies, though not all (Widom et al., 2007; Fergusson et al., 2008; Agorastos et al., 2014), have shown that CTEs are associated with social support and behavioral factors in adulthood. The results of this study support this. Higher alcohol use among victims of CTEs may reflect the need to reduce feelings of isolation and loneliness (Widom et al., 1995; Widom and Hiller-Sturmhofel, 2001). In this way, alcohol use may act as a mediator in the association between social support factors, and health and well-being in adulthood. Future studies should address this question.

In this dataset, we observed that other structural conditions, such as gender and socioeconomic adversities in childhood are important to health and well-being in adulthood. However, they do not play a moderating role in the association between CTE combinations and adult health and well-being. Some studies have suggested that socioeconomic factors and CTEs may interact to increase one's vulnerability to traumatic experiences in childhood (Schilling and Christian, 2014), but we found no statistically significant multiplicative interaction between CTE combinations and any of the confounding variables (data not shown). Previous studies have reported statistically significant multiplicative interactions between adversities in childhood and adversities in adulthood in predicting certain negative health outcomes (Kessler and Magee, 1994; Mock and Arai, 2011; Aas et al., 2014; Sheikh et al., 2014), whereas we did not find convincing evidence in this study (data not shown). Some evidence (Horwitz et al., 2001; Chartier et al., 2010; Axinn et al., 2013; De Bellis and Zisk, 2014; Horan and Widom, 2014), though not all (Greenfield and Marks, 2009, 2010; Springer, 2009; Greenfield et al., 2011), suggests that gender may be a moderator of CTEs, or of behavioral factors (Widom and White, 1997). As we found no such evidence in our sample, estimates are provided for men and women combined. Similarly, no statistically significant multiplicative interaction with age was observed in our sample (data not shown).

Most previous studies (McLeod, 1991; Harper et al., 2002; Luo and Waite, 2005; Fors et al., 2009; Gibb et al., 2012; Morgan et al., 2014; Monnat and Chandler, 2015; Pavela and Latham, 2015), though not all (Danese et al., 2009; Park et al., 2013; Sheikh et al., 2014), have suggested that adult socioeconomic status (ASES) mediates the effect of childhood adversities on indicators of health in adulthood. The crude associations between CTE combinations and ASES (measured using the variables: education, having full-time occupation, income, and subjective social status) did not show a clear pattern (data not shown). Similarly, the association between CTEs, and subjective social status in adulthood was not statistically significant (data not shown). These are key assumptions that must be satisfied before assessing mediation (Judd and Kenny, 1981; Baron and Kenny, 1986). Consequently, including ASES in the regression models (adjusted for confounding variables, and social support and behavioral factors) barely attenuated the estimates of CTEs (Sheikh, 2015), and ASES was thus not included in the final analyses. If the models are only adjusted for ASES (and not social support and behavioral factors), the attenuation in estimates may be due to the association between ASES and social support and behavioral factors. In this case, ASES may serve as a surrogate mediator, and the estimates will be attenuated depending on the strength of the correlation between ASES and social support and behavioral factors. It is plausible that in the absence (or mismeasurement) of social support and behavioral factors in the models, previous studies concluded that ASES mediates the association between childhood adversity and health in adulthood. One possible reason for ASES not being a mediator in our study sample may be the Norwegian welfare state, and the presence of free education at all levels. This means that even respondents with disadvantaged backgrounds may still obtain the necessary education to build a career. Moreover, respondents with severe traumatic experiences and difficulty in keeping a stable job or in landing a high-income job due to behavioral or psychological problems may still not be poor in absolute terms, as the welfare benefits provide for the basic amenities in life.

It is plausible that different mechanisms apply to different respondents in the sample. For instance, there may be a subset of the sample, for which the mediators do not operate by the hypothesized mechanism. For example, previous research has shown that children growing up in a violent environment have high levels of life satisfaction and optimism (Veronese et al., 2012b). Other evidence suggests that some children are not affected by psychological and social problems despite traumatic experiences (Luthar et al., 2000). This “resilience” may be explained by genetic factors (Lykken and Tellegen, 1996; Frey, 2011), positive emotions (Veronese et al., 2012a,c), and contextual factors (Fergusson and Lynskey, 1997; Veronese et al., 2012b; Veronese and Castiglioni, 2015), that enable the children to develop a resilient identity. This may be detected by the significance and magnitude of product terms involving socio-demographic variables (where socio-demographic variables may serve as a proxy for genetic and contextual factors). However, since the empirical tests of multiplicative interactions involving multiple factors are notoriously underpowered (Marshall, 2007), the possibility of moderation cannot be ruled out. On the contrary, other evidence suggests that physical and psychological abuse in childhood has consequences on a molecular level (thus, cannot be manipulated by the subject), even when culturally or contextually acceptable (Hecker et al., 2016).

This study has some limitations. An important assumption when assessing mediation (indirect effects) is temporality (Nguyen et al., 2015). The mediators must precede the outcomes, and the exposure must precede the mediators. We acknowledge that the temporality between mediators and mental health, health, and well-being cannot be determined empirically in this study. There may be some reverse causation, as those who are unhealthy are likely to experience problems in interpersonal relations. Therefore, the present study cannot determine whether the factors in adulthood are the cause, or the consequence of poor health/low well-being.

The retrospective account of CTEs may be subject to recall bias (Cohen et al., 1997; Gilbert, 2006), deleterious effects on memory among those suffering from clinical states (such as anxiety and depression), and retrieval bias among those with clinical states (Brewin et al., 1993; Williams, 1994; Goldsmith et al., 2009; Saunders and Adams, 2014). It could be argued that unhealthy individuals maybe more likely to report or recall CTEs. However, a review of the evidence suggests that these biases should be fairly low (Brewin et al., 1993).

The cut-offs used in this manuscript are aimed to separate the “*most* unhealthy”/”*lowest* well-being” from the rest. We performed all analyses using the alternative cut-offs (“*perfectly* healthy/*highest* well-being” *vs*. the rest) and the results were consistent in the same direction (see Online Supplementary Material). We acknowledge that the measures of mental health, health, and well-being were dichotomized for mediation analysis, and thus lost much of their variation. All three dependent variables did not fulfill the normality assumptions for the OLS regression model that are needed for hypothesis testing. An alternative solution would have been to use quantile regression models, but so far no methodological approach for assessing mediation with quantile regression has been established in the literature. However, we assessed mediation with alternative cut-offs, and the results remained consistent in the same direction (see Online Supplementary Material).

It should be noted that the magnitude of RRs also reflects the way the cut-offs for the outcome variables are made. The cut-off for the SCL-10 was based on prior research (Strand et al., 2003; Kvamme et al., 2011). It is likely that the RRs would have been different in magnitude had we used a different cut-off. However, the direction of the effects proved to remain the same for other cut-offs that we tested (see Online Supplementary Material).

Assessing mediation with the measure of *proportion mediated* (Susser, 1973; Szklo and Nieto, 2000; Kaufman et al., 2004) is not without limitations (MacKinnon, 2008). The CIs for this measure are often wide, and have no limit. This is particularly apparent when the effect size (total effect) is small *or* when the direct effect is very close to null (*RR*_Direct Effect_ ≈ 1.00). The confidence interval of *proportion mediated (% attenuation)* may include many values far greater than 100%, simply because many random samples from the study sample, may estimate the total effect *or* the direct effects less than 1.00. Similarly, the *proportion mediated* measure does not provide a valid interpretation when there is inconsistent mediation, such that the direct and indirect effects have opposite signs; potentially arising from selection bias by list-wise deletion. In the case of mental health, estimates for Ps_0_Ph_1_D_1_ demonstrate this problem, as the estimate for direct effect increased (as compared to total effects) after including the mediators in the models (Table 11). This was not the case when multiple imputed dataset was used for analysis (Table 12). Still, for what it is worth; *proportion mediated* provides a simple explanation of the indirect effect in terms of percentages.

We acknowledge that the three measures of mental health, health, and well-being used in this study were not commonly used in the previous psychology literature. We performed multiple statistical tests to address the research questions, and this may increase the probability of obtaining a type I error. Previous studies have shown that significance probability should not be adjusted (for instance, using *p* < 0.01, instead of *p* < 0.05) to reduce the chance of type I error, as doing so may increase the probability of type II error (Rothman, 1990; Feise, 2002).

We classified the experience of having a close family member using alcohol or drugs in such a way that it caused worry as “substance abuse distress.” We acknowledge that this classification may be misleading since the variable probably represents the *worry* of witnessing problematic drinking or drug use by a family member. It cannot be established empirically from the data whether the *worry* was enough to be established as *distress*, as this is a theoretical question. The results from the imputed dataset (Tables 6, 12) showed that substance abuse distress was significantly associated with mental health and well-being, but not health. This implies that the effect of this *worry*, classified as *substance abuse distress*, is long-term, at least for mental health and subjective well-being. Whether this specific type of *distress* merits classification as a CTE is beyond the scope of this paper. The strengths of this study lie in its large representative population sample, its estimates of different types of CTEs, and the use of three multi-item instruments for mental health, health, and well-being.

This study contributes to the growing literature (Dube et al., 2001; Baker et al., 2009; Fors et al., 2009; Mock and Arai, 2011; Landes et al., 2014; Raposo et al., 2014; Sheikh et al., 2014; van Nierop et al., 2014; Gilman et al., 2015; May-Ling et al., 2015; Pavela and Latham, 2015) on the assessment of lifetime pathways from childhood adversity to mental health, health, and well-being in adulthood. Using Shapley decomposition for dissimilarity index and *R*^2^, we showed the relative contribution of socio-demographic factors, CSES, CTEs, social support and behavioral factors for mental health, health, and well-being. We used the “difference method” approach to assess mediation. The estimates were adjusted for potential confounding variables, and the multiplicative interaction between the types of CTEs was considered. Our findings suggest that childhood traumatic experiences increase the risk of being unhealthy and having a low level of well-being, and that some of this effect is mediated by social support and behavioral factors in adulthood.

## Ethics Statement

The Tromsø Study has been approved by the Regional Committee for Medical and Health Research Ethics, the Data Inspectorate and the Norwegian Directorate of Health. Written informed consent was obtained from all individual participants included in the study.

## Author contributions

This work was completed as part of MS's PhD. MS planned the study, performed statistical analyses, data interpretation, developed the theory and drafted the manuscript. BA and JO critically commented on the manuscript.

## Funding

This work was funded by the University of Tromsø, Norway.

### Conflict of interest statement

The authors declare that the research was conducted in the absence of any commercial or financial relationships that could be construed as a potential conflict of interest.
