# Supplementary material for: Clarifying Associations between Childhood Adversity, Social Support, Behavioral Factors, and Mental Health, Health, and Well-Being in Adulthood: A Population-Based Study
Source: Front Psychol. 2016 May 25;7:727. doi: 10.3389/fpsyg.2016.00727 (PMC4879780; doi:10.3389/fpsyg.2016.00727)
Supplement: Supplementary file 1 [file DataSheet1.docx]

**Online Supplementary Material**

The cut-offs used in this Online Supplementary Material are aimed to separate the ‘*perfectly* healthy/*highest* well-being’ from the rest, in classifying health and well-being variables.

**Classification of health and well-being**

**Health (EQ-5D)**

Health was assessed in the study questionnaire by the EQ-5D generic measure of health-related quality of life. The EQ-5D includes five health dimensions: mobility, self-care, usual activities, pain/discomfort, and anxiety/depression (The EuroQol Group, 1990). Each health dimension has three levels: 1) no problems, 2) some problems, and 3) unable or extreme problems. A composite EQ-5D binary variable was constructed for this supplementary analyses by classifying all respondents who ticked level 1 for all five health dimensions as healthy (Y=0), and all others as relatively unhealthy (Y=1).

**Subjective well-being (SWLS)**

Well-being was measured by the response to the first three items on the satisfaction with life scale (SWLS) (Diener, 1985; Oishi, 2006). These were: ‘In most ways my life is close to my ideal’, ‘The conditions of my life are excellent’, and ‘I am satisfied with my life’. Respondents rated these statements using a 7-point scale ranging from completely disagree (1) to completely agree (7). A composite binary well-being variable was constructed by classifying those who responded with a 6 or 7 on all three items as having a high level of well-being (Y=0), and all others as having relatively low level of well-being (Y=1). The cut-off points for classifying health, and well-being are somewhat arbitrary, but are in agreement with previous studies (Sheikh, 2014, 2015; Sheikh, Abelsen, & Olsen, 2014).

***Statistical analysis***

All analyses were conducted using Stata version 13 and R (ver. 3.1.2). Associations were considered significant at *p* <0.05. The association between the different combinations of traumatic experiences in childhood and the selected mediators, and the association between the mediators and the different measures of health and well-being in adulthood was assessed with correspondence analysis (M. Greenacre, 2007; Michael Greenacre, 2010) (Figures S3 and S4). Correspondence analysis is a multivariate descriptive technique that decomposes the chi-square statistic. Figures representing a two-dimensional joint space (biplots) illustrate the association between two groups of variables (traumatic experiences with mediators, and mediators with outcomes). Categories with similar distributions are closer together, while the categories with dissimilar distributions are farther apart.

Poisson regression analysis with robust error variance (Barros & Hirakata, 2003; Zou, 2004) was used to estimate the relative risk (RR and 95% confidence intervals (CIs)) of being unhealthy, and having a low well-being in adulthood dependent on traumatic experiences in childhood.

**Results:**

Table S1 presents the general characteristics of the study sample with n and % in the dataset. The distributions of health and well-being were similar in the un-imputed dataset (excluding those with missing values) and the imputed dataset with MI (Table S1).

**Relative contribution of CSES, socio-demographic factors, traumatic experiences in childhood, social support, and behavioural factors to health and well-being**

Table S2 presents the average marginal contribution for health and well-being, of all explanatory variables used in this study. The Shapley decomposition of dissimilarity index shows that among all the variables considered, gender (25.24%) explained most of the variation in health, while childhood financial conditions (25.88%) explained most of the variation in well-being.

Among all the indicators of childhood adversities, childhood financial conditions (9.64%) was most important for health in adulthood, followed by mother’s education (6.33%), psychological abuse (6.15%), father’s education (4.64%), physical abuse (3.26%), and substance abuse distress (1.05%). Furthermore, for well-being, childhood financial conditions (25.88%) was most important, followed by psychological abuse (8.05%), physical abuse (5.31%), and substance abuse distress (5.21%), mother’s education (2.96%), and father’s education (2.11%). However, if the traumatic experiences in childhood are considered together, they were relatively more important for health (20.61% vs 10.46% ), and well-being (30.95% vs 18.57%) than traumatic experiences in childhood (Table S3).

Similarly, if both the behavioural factors are considered together, they (15.6%) were relatively more important for health than social support factors (14.73%). On the other hand, social support factors were relatively more important for well-being (37.49%).

**Associations between traumatic experiences in childhood, and psycho-social and behavioral factors**

A biplot of the association between traumatic experiences in childhood (in red) and mediators (in blue) explained 93.2% of the total variation in the cross-tabulation between these variables (Figure S3). Having no traumatic experience (Ps_0_Ph_0_D_0_) was clearly separated from having any combination of traumatic experience and was closely associated with having friends to talk to and get support from, being a former or never daily smoker, and low alcohol consumption. In contrast, being exposed to any combination of traumatic experiences in childhood was associated with being a daily smoker, not having friends to talk to or get support from, and high alcohol consumption (Figure S3).

**Interaction between traumatic experiences**

Figures S1 and S2 show the interaction between traumatic experiences in childhood. The influence of physical abuse on well-being changed depending on exposure to psychological abuse (Figure S1). Similarly, the influence of substance abuse distress on well-being changed depending on exposure to physical abuse in childhood (Figure S2).

**Associations between social support, behavioural factors, and health and well-being**

A biplot of the association between mediators (in blue) and health and well-being (in red) explained 99.1% of the total variation in the cross-tabulation between these variables (Figure S4). Healthy respondents and those with a high level of well-being were clearly separated from unhealthy respondents and those with a low level of well-being and being mentally unhealthy was associated with having no friends to get support from and talk to, and drinking 10 or more units of alcohol. Being unhealthy and having a low level of well-being was associated with drinking 5-9 units of alcohol and being a smoker. Finally, being healthy and having a high level of well-being was associated with being a never smoker, having friends to talk to and get support from, and low alcohol consumption.

Table S4 shows the distribution of mediators by health and well-being. The crude distributions between mediators and health and well-being showed that most mediators were significantly associated with health and well-being in adulthood (Table S4). The crude associations were in the predicted direction, i.e., those who were current or former smokers, had no friends to get support from and talk to, and drank five or more units of alcohol whenever they drank, tend to be unhealthy and have a low level of well-being (Table S4).

Table S5 shows the association between mediators and health and well-being. Both the complete-case analysis (excluding missing), and the imputed dataset analysis are presented. Analyses conducted on the imputed dataset showed that the estimates for health and well-being were mostly similar to those in the complete-case dataset. Having no instrumental support, no emotional support, higher alcohol use, and being a daily smoker was associated (*p*<0.05) with an increased risk of being unhealthy, and a low level of well-being (Table S5).

Among the two indicators of social support, having *no* instrumental support led to a higher risk for being unhealthy (RR=1.19 for instrumental support vs RR=1.13 for emotional support), while having *no* emotional support led to a higher risk for having a low level of well-being (RR=1.22 for emotional support vs RR=1.15 for instrumental support). The test for linear trend (*p*<0.05) showed that increased alcohol use was associated with being unhealthy and having a low level of well-being (Table S5).

**Association between different combinations of traumatic experience in childhood and health and well-being**

Table S6 presents the estimates for the risk of being unhealthy, and having a low level of well-being with exposure to different combinations of traumatic experience in childhood. Three estimates are presented: unadjusted, adjusted for confounding variables, and adjusted for confounding variables and mediators. The test of linear trend (*p*<0.05) shows that *trauma frequency* was associated with a higher risk of being unhealthy and having a low level of well-being. However, the estimates showed that the association may not be linear. The risk for being unhealthy by exposure to any *two* types of traumatic experiences in childhood was lower than exposure to any *one* traumatic experience. Similarly, the risk of having a low level of well-being (RR=1.14) remained almost the same for being exposed to any *one*, any *two*, or all *three* traumatic experiences in childhood (Table S6). Compared to those exposed to substance abuse distress, those exposed to both psychological and physical abuse had a higher risk of being unhealthy (RR=1.13, 95% CI: 1.04-1.23). Similarly, compared to those exposed to substance abuse distress, those exposed to psychological abuse had an 8% (RR=1.08, 95% CI: 1.01-1.16) higher risk of being unhealthy (Table S6).

Most comparisons presented in Table S6 were not statistically significant (*p*>0.05); however, generally the estimates were attenuated after adjusting for mediators (particularly for *trauma frequency* variable). This shows that despite the large sample size, there was no significant difference between the three types of traumatic experiences for most comparisons.

**Total effects: the association between traumatic experiences in childhood, and health and well-being in adulthood**

Those exposed to traumatic experiences in childhood tend to be unhealthy and have a low level of well-being (Table S3). Table S7 and S8 presents the estimates for the risk of being unhealthy, and having a low level of well-being with exposure to traumatic experiences in childhood (reference: not exposed to any of the three traumatic experiences). Three estimates are presented: crude (unadjusted), adjusted for confounding variables (total effects), and adjusted for confounding variables and mediators (direct effects). The reference category is those not exposed to any traumatic experiences in childhood. The unadjusted (crude) associations show that being exposed to all the combinations of traumatic experiences in childhood significantly (*p*<0.05) increased the risk of being unhealthy and having a low level of well-being (Table S7). After adjustment for confounding variables, there was a significant, increased risk of being unhealthy and of having a low level of well-being for most combinations of traumatic experiences in childhood (Table S7). The exceptions was; being exposed to substance abuse distress only for health (Ps_0_Ph_0_D_1_).

The Shapley decomposition of dissimilarity index for total effects showed that exposure to psychological abuse only (Ps_1_Ph_0_D_0_) and exposure to both psychological and physical abuse (Ps_1_Ph_1_D_0_) explained the largest proportion in both health and well-being, as compared to other trauma combinations in unadjusted models and models adjusted for confounding variables (Table S7). This is consistent with results in Table S2.

**Direct effects: the association between traumatic experiences in childhood, and health and well-being in adulthood, when controlling for social support and behavioural factors**

Table S7 and S8 presents the direct effects (adjusted for confounding variables and mediators). The reference category is those not exposed to any traumatic experiences in childhood. The direct effects show that for both health and well-being, exposure to most combination of traumatic experiences in childhood significantly (*p*<0.05) increased the risk of being unhealthy. The results from the imputed dataset showed that exposure to psychological abuse only (Ps_1_Ph_0_D_0_), exposure to both psychological and physical abuse (Ps_1_Ph_1_D_0_), exposure to both psychological abuse and substance abuse distress (Ps_1_Ph_0_D_1_), exposure to both physical abuse and substance abuse distress (Ps_0_Ph_1_D_1_), and exposure to all three traumatic experiences (Ps_1_Ph_1_D_1_) increased the risk of being unhealthy (Table S7). Results from the imputed dataset showed that exposure to physical abuse only (Ps_0_Ph_1_D_0_) was associated with a 13% higher risk of being unhealthy (RR=1.13, 95% CI: 1.05-1.22) (Table S8). For well-being, all combinations of traumatic experiences in childhood significantly increased the risk of having a low level of well-being (Table S7). Those exposed to psychological abuse only (Ps_1_Ph_0_D_0_) in childhood had a 22% increased risk of being unhealthy, and a 15% increased risk of having a low level of well-being (Table S7). Those exposed only to physical abuse (Ps_0_Ph_1_D_0_) in childhood had a 20% increased risk of having a low level of well-being, whereas those exposed only to substance abuse distress (Ph_0_Ps_0_D_1_) in childhood had an increased risk of 11% (Table S7). Those exposed to all three types of childhood traumatic experiences (Ps_1_Ph_1_D_1_) had a 38% increased risk of being unhealthy, and a 16% increased risk of having a low level of well-being (Table S7).

The Shapley decomposition of dissimilarity index for direct effects showed that, for models adjusted for mediators, the exposure to psychological abuse only (Ps_1_Ph_0_D_0_) and exposure to both psychological and physical abuse (Ps_1_Ph_1_D_0_) explained most of the variation in health (EQ-5D) (Table S7). However, for well-being, exposure to physical abuse only (Ps_0_Ph_1_D_0_) and exposure to both psychological and physical abuse (Ps_1_Ph_1_D_0_) explained most of the variation in well-being (Table S7).

Analysis conducted on the imputed dataset (Table S8) show that the estimates for direct effects (and 95% CIs) for health and well-being were nearly the same as those in the complete-case dataset presented in Table S7. In most of the models, there was a statistically significant association between childhood traumatic experiences, and health and well-being after adjusting for mediators. Furthermore, some of the models that were not statistically significant (*p*≥0.05) in the complete-case dataset (Table S7) were statistically significant (*p*<0.01) in the imputed dataset (Table S8), and were in the same direction.

**Table S1. Proportion (%) of health and well-being in the un-imputed dataset, and in the imputed dataset with multiple imputation (n=12,981)**

|  | | Un-imputed dataset | Imputed dataset |
| --- | --- | --- | --- |
|  |  | % | % |
| **Health and well-being** |  |  |  |
| - Health (EQ-5D) | Healthy | 44.6 | 43.2 |
|  | Unhealthy | 55.4 | 56.8 |
| - Well-being (SWLS) | High | 36.4 | 36.3 |
|  | Low | 63.6 | 63.7 |

EQ-5D: Health was assessed by the EQ-5D generic measure of health-related quality of life. SWLS: Well-being was measured by the satisfaction with life scale (SWLS)

**Table S2. Relative contribution of CSES, socio-demographic factors, traumatic experiences in childhood, social support and behavioural factors for health (EQ-5D), and subjective well-being (SWLS)**

| **Explanatory variables** | **Shapley decomposition of dissimilarity index (% explained)** | |
| --- | --- | --- |
|  | **Health (EQ-5D)** | **Well-being (SWLS)** |
|  | **%** | **%** |
| Psychological abuse | 6.15 | 8.05 |
| Physical abuse | 3.26 | 5.31 |
| Substance abuse distress | 1.05 | 5.21 |
| Age | 13.33 | 1.40 |
| Sex | 25.24 | 1.33 |
| Mother’s education | 6.33 | 2.96 |
| Father’s education | 4.64 | 2.11 |
| Childhood financial conditions | 9.64 | 25.88 |
| Instrumental support^a^ | 7.98 | 15.86 |
| Emotional support^a^ | 6.75 | 21.63 |
| Alcohol use (units)^b^ | 1.91 | 5.21 |
| *Daily smoking*^b^ |  |  |
| Never smoker (ref) | Ref | Ref |
| Past smoker | 4.81 | 1.76 |
| Daily smoker | 8.88 | 3.28 |

^a^Social support factors were measured by instrumental and emotional support. Instrumental support: Do you have enough friends who can give you help and support when you need it? (yes, no); Emotional support: Do you have enough friends you can talk confidentially with? (yes, no)

^b^Behavioural factors were measured by two questions: Do you/did you smoke daily? (yes, now; yes, previously; never (ref)); How many units of alcohol (a beer, a glass of wine or a drink) do you usually drink when you drink alcohol? (1-4, 5-6, 7-9, 10 or more).

EQ-5D: Health was assessed by the EQ-5D generic measure of health-related quality of life

SWLS: Well-being was measured by the satisfaction with life scale (SWLS)

**Figure S1: Effect of physical abuse on subjective well-being (SWLS) in adulthood, by psychological abuse in childhood (logit scale).**

.4

.6

.8

1

1.2

**Well-being (SWLS)**

0

.2

.4

.6

.8

1

**Physical abuse in childhood**

Psychological abuse = No

Psychological abuse = Yes

Physical abuse by psychological abuse

**Figure S2: Effect of physical abuse on subjective well-being (SWLS) in adulthood, by substance abuse distress in childhood (logit scale).**

Dimension 1 (83.3%)

Dimension 2 (9.9%)

-0.6

-0.4

-0.2

0.0

-0.05

0.00

0.05

0.10

0.15

0.20

Support Friends: No

Support Friends: Yes

Talk Friends: No

Talk Friends: Yes

Smoker

Former smoker

Never smoker

A:1-4 units

A:5-6 units

A:7-9 units

A:10 or more units

Ps_0_Ph_0_D_0_

Ps_1_Ph_0_D_0_

Ps_0_Ph_1_D_0_

Ps_0_Ph_0_D_1_

Ps_1_Ph_1_D_0_

Ps_1_Ph_0_D_1_

Ps_0_Ph_1_D_1_

Ps_1_Ph_1_D_1_

**Figure S3: Biplot showing the association between traumatic experiences in childhood (in red) with mediating factors in adulthood (in blue).**

Alcohol units; A:1-4, A: 5-6, A:7-9, A:10 or more; Ps_0_Ph_0_D_0_: Not exposed to psychological abuse, physical abuse, or distress in childhood. Ps_0_Ph_0_D_0_: Not exposed to psychological abuse, physical abuse and substance abuse distress in childhood. Ps_1_Ph_0_D_0_: Exposed to psychological abuse but not physical abuse and substance abuse distress. Ps_0_Ph_1_D_0_: Exposed to physical abuse but not psychological abuse and substance abuse distress. Ps_0_Ph_0_D_1_: Exposed to substance abuse distress, but not psychological abuse and physical abuse. Ps_1_Ph_1_D_0_: Exposed to both psychological and physical abuse but not substance abuse distress. Ps_1_Ph_0_D_1_: Exposed to both psychological abuse and substance abuse distress but not physical abuse. Ps_0_Ph_1_D_1_: Exposed to both physical abuse and substance abuse distress but not psychological abuse. Ps_1_Ph_1_D_1_: Exposed to psychological abuse, physical abuse, and substance abuse distress.

**Figure S4: Biplot showing the association between psycho-social and behavioural mediators in adulthood (in blue) and health and well-being in adulthood (in red).**

Dimension 1 (96.9%)

Dimension 2 (2.2%)

-0.6

-0.4

-0.2

0.0

-0.10

-0.05

0.00

0.05

0.10

0.15

Support friends: No

Support friends:Yes

Talk friends: No

Talk friends:Yes

Smoker

Former smoker

Never Smoker

A:1-4

A: 5-6

A: 7-9

A: 10 or more

Well-being: High

Well-being: Low

SCL-10: Healthy

SCL-10: Unhealthy

EQ-5D: Healthy

EQ.5D: Unhealthy

Alcohol units; A:1-4, A: 5-6, A:7-9, A:10 or more.

**Table S3. Distribution (%) of health and well-being by exposure to traumatic experiences in childhood**

|  |  | Ps_0_Ph_0_D_0_ | Ps_1_Ph_0_D_0_ | Ps_0_Ph_1_D_0_ | Ps_0_Ph_0_D_1_ | Ps_1_Ph_1_D_0_ | Ps_1_Ph_0_D_1_ | Ps_0_Ph_1_D_1_ | Ps_1_Ph_1_D_1_ | Test statistic | *p* |
| --- | --- | --- | --- | --- | --- | --- | --- | --- | --- | --- | --- |
|  |  | n=10,907 | n=525 | n=230 | n=643 | n=393 | n=106 | n=44 | n=133 |  |  |
| **Health & well-being** |  |  |  |  |  |  |  |  |  |  |  |
| Health (EQ-5D) | Healthy | 46.2 | 35.8 | 39.9 | 43.6 | 30.5 | 28.2 | 32.6 | 26.5 | χ² (7) = 90.44 | *p*<0.01 |
|  | Unhealthy | 53.8 | 64.2 | 60.1 | 56.4 | 69.5 | 71.8 | 67.4 | 73.5 |  |  |
| Well-being (SWLS) | High | 38.9 | 26.6 | 22.7 | 30.0 | 21.7 | 15.7 | 23.3 | 23.1 | χ² (7) = 138.75 | *p*<0.01 |
|  | Low | 61.1 | 73.4 | 77.3 | 70.0 | 78.3 | 84.3 | 76.7 | 76.9 |  |  |

Ps_0_Ph_0_D_0_: Not exposed to psychological abuse, physical abuse and substance abuse distress in childhood. Ps_1_Ph_0_D_0_: Exposed to psychological abuse but not physical abuse and substance abuse distress. Ps_0_Ph_1_D_0_: Exposed to physical abuse but not psychological abuse and substance abuse distress. Ps_0_Ph_0_D_1_: Exposed to substance abuse distress, but not psychological abuse and physical abuse. Ps_1_Ph_1_D_0_: Exposed to both psychological and physical abuse but not substance abuse distress. Ps_1_Ph_0_D_1_: Exposed to both psychological abuse and substance abuse distress but not physical abuse. Ps_0_Ph_1_D_1_: Exposed to both physical abuse and substance abuse distress but not psychological abuse. Ps_1_Ph_1_D_1_: Exposed to psychological abuse, physical abuse, and substance abuse distress.

EQ-5D: Health was assessed by the EQ-5D generic measure of health-related quality of life SWLS: Well-being was measured by the satisfaction with life scale (SWLS)

**Table S4. Distribution (%) of mediators by health (EQ-5D) and subjective well-being (SWLS)**

|  | |  | Health (EQ-5D) | |  | Well-being (SWLS) | | |
| --- | --- | --- | --- | --- | --- | --- | --- | --- |
|  | |  | Unhealthy | Healthy |  | Low | High | Test statistic |
|  | |  | n=6,437 | n=5,176 | Test statistic | n=6,843 | n=3,917 |  |
| **Mediators** |  | |  |  |  |  |  |  |
| Instrumental support^a^ | Yes | | 52.8 | 47.2 | χ² (1) = 208.35* | 60.7 | 39.3 | χ² (1) = 251.55* |
|  | No | | 75.0 | 25.0 |  | 85.5 | 14.6 |  |
| Emotional support^a^ | Yes | | 53.2 | 46.9 | χ² (1) = 131.06* | 60.3 | 39.7 | χ² (1) = 284.26* |
|  | No | | 69.4 | 30.6 |  | 84.3 | 15.7 |  |
| Daily smoking ^b^ | Never | | 49.2 | 50.8 | χ² (2) = 120.69* | 60.1 | 39.9 | χ² (2) = 41.59* |
|  | Previously | | 57.3 | 42.7 |  | 64.9 | 35.1 |  |
|  | Yes | | 62.4 | 37.5 |  | 67.9 | 32.1 |  |
| Alcohol units ^b, c^ | 1-4 | | 53.9 | 46.2 | χ² (3) = 7.30 | 62.7 | 37.3 | χ² (3) = 29.77* |
|  | 5-6 | | 54.0 | 46.0 |  | 69.7 | 30.3 |  |
|  | 7-9 | | 61.0 | 39.0 |  | 70.5 | 29.5 |  |
|  | 10 or more | | 65.6 | 34.4 |  | 84.8 | 15.3 |  |

**P*<0.05

^a^Social support factors were measured by instrumental and emotional support. Instrumental support: Do you have enough friends who can give you help and support when you need it? (yes, no); Emotional support: Do you have enough friends you can talk confidentially with? (yes, no)

^b^Behavioural factors were measured by two questions: Do you/did you smoke daily? (yes, now; yes, previously; never); How many units of alcohol (a beer, a glass of wine or a drink) do you usually drink when you drink alcohol? (1-4, 5-6, 7-9, 10 or more).

EQ-5D: Health was assessed by the EQ-5D generic measure of health-related quality of life

SWLS: Well-being was measured by the satisfaction with life scale (SWLS)

^c^Test for linear trend *p*<0.05

**Table S5. Association between mediators, and health (EQ-5D) and subjective well-being (SWLS)**

|  |  | **Complete-case analysis (excluding missing)** | | **Imputed dataset with multiple imputation (n=12,981)** | |
| --- | --- | --- | --- | --- | --- |
| Mediators^a, b^ | | Unadjusted | Adjusted^g^ | Unadjusted | Adjusted^g^ |
|  |  | RR (95% CI) | RR (95% CI) | RR (95% CI) | RR (95% CI) |
|  |  | **Health (EQ-5D)** | | | |
| Instrumental support^a^ | No^d^ | **1.42 (1.37-1.47)** | **1.18 (1.11-1.26)** | **1.40 (1.35-1.45)** | **1.19 (1.13-1.25)** |
| Emotional support^a^ | No^d^ | **1.30 (1.25 1.36)** | **1.16 (1.09-1.23)** | **1.29 (1.25-1.34)** | **1.13 (1.09-1.19)** |
| Alcohol use (units) ^b, h^ | 5-6^e^ | 1.00 (0.94-1.07) | **1.13 (1.05-1.21)** | 0.98 (0.92-1.04) | **1.11 (1.04-1.18)** |
|  | 7-9^e^ | **1.13 (1.01-1.27)** | **1.31 (1.16-1.47)** | 1.10 (0.99-1.23) | **1.30 (1.17-1.44)** |
|  | 10 or more^e^ | **1.22 (1.01-1.46)** | **1.37 (1.12-1.67)** | 1.17 (0.99-1.40) | **1.33 (1.11-1.59)** |
| Daily smoking ^b^ | Previous smoker^f^ | **1.16 (1.12-1.21)** | **1.16 (1.11-1.22)** | **1.15 (1.11-1.19)** | **1.13 (1.09-1.17)** |
|  | Current smoker^f^ | **1.27 (1.22-1.33)** | **1.25 (1.18-1.31)** | **1.25 (1.20-1.31)** | **1.22 (1.17-1.27)** |
|  |  | **Well-being (SWLS)** | | | |
| Instrumental support^a^ | No^d^ | **1.41 (1.37-1.45)** | **1.16 (1.11-1.21)** | **1.38 (1.34-1.42)** | **1.15 (1.11-1.20)** |
| Emotional support^a^ | No^d^ | **1.40 (1.36-1.44)** | **1.23 (1.18-1.29)** | **1.38 (1.34-1.42)** | **1.22 (1.17-1.27)** |
| Alcohol use (units) ^b, h^ | 5-6^e^ | **1.11 (1.06-1.17)** | **1.10 (1.04-1.16)** | **1.10 (1.05-1.16)** | **1.09 (1.03-1.14)** |
|  | 7-9^e^ | **1.12 (1.03-1.23)** | **1.11 (1.01-1.22)** | **1.11 (1.01-1.21)** | 1.08 (0.98-1.18) |
|  | 10 or more^e^ | **1.35 (1.21-1.51)** | **1.25 (1.09-1.42)** | **1.31 (1.17-1.48)** | **1.24 (1.10-1.40)** |
| Daily smoking ^b^ | Previous smoker^f^ | **1.08 (1.04-1.12)** | **1.09 (1.04-1.13)** | **1.07 (1.03-1.10)** | **1.09 (1.05-1.13)** |
|  | Current smoker^f^ | **1.13 (1.09-1.17)** | **1.06 (1.02-1.09)** | **1.12 (1.08-1.17)** | **1.05 (1.01-1.08)** |

All significant associations (*p*<0.05) are in bold.

^a^Social support factors were measured by instrumental and emotional support. Instrumental support: Do you have enough friends who can give you help and support when you need it? (yes, no); Emotional support: Do you have enough friends you can talk confidentially with? (yes, no)

^b^Behavioral factors were measured by two questions: Do you/did you smoke daily? (yes, now; yes, previously; never); How many units of alcohol (a beer, a glass of wine or a drink) do you usually drink when you drink alcohol? (1-4, 5-6, 7-9, 10 or more).

EQ-5D: Health was assessed by the EQ-5D generic measure of health-related quality of life. SWLS: Well-being was measured by the satisfaction with life scale (SWLS)

^d^Reference: Yes.

^e^Reference: 1-4 units.

^f^Reference: Never.

^g^Adjusted for psychological abuse, physical abuse, substance abuse distress, confounding variables and other mediators

^h^Test for linear trend *p*<0.05

**Table S6. Association between traumatic experiences, health (EQ-5D), and subjective well-being (SWLS) (n=12,981)**

|  |  | **Imputed dataset with multiple imputation (n=12,981)** | | |
| --- | --- | --- | --- | --- |
| Traumatic experiences |  | Unadjusted | Adjusted^a^ | Adjusted^e^ |
|  |  | RR (95% CI) | RR (95% CI) | RR (95% CI) |
|  | n | **Health (EQ-5D)** | | |
| Trauma frequency (reference: not exposed)^f^ | 10907 | Ref | Ref ^a^ | Ref |
| - Exposed to any one traumatic experience | 525 | **1.17 (1.09-1.25)** | **1.22 (1.14-1.30)** | **1.20 (1.12-1.28)** |
| - Exposed to any two traumatic experiences | 1416 | **1.13 (1.09-1.18)** | **1.17 (1.12-1.22)** | **1.13 (1.08-1.18)** |
| - Exposed to all three traumatic experiences | 133 | **1.33 (1.20-1.47)** | **1.38 (1.25-1.53)** | **1.33 (1.20-1.46)** |
| Psychological abuse vs physical abuse (ref) | 1431 | 0.97 (0.90-1.05) | 0.95 (0.89-1.02)^d^ | 0.97 (0.90-1.04)^i^ |
| Psychological abuse vs substance abuse distress (ref) | 1844 | **1.09 (1.01-1.16)** | **1.08 (1.01-1.16)**^c^ | **1.08 (1.01-1.16)^h^** |
| Physical abuse vs substance abuse distress (ref) | 1549 | **1.08 (1.00-1.17)** | 1.03 (0.95-1.11)^b^ | 1.02 (0.94-1.10)^g^ |
| Psychological abuse and physical abuse vs substance abuse distress (ref) | 1319 | **1.14 (1.05-1.23)** | **1.16 (1.07-1.26)^a^** | **1.13 (1.04-1.23)** |
| Psychological abuse and distress vs physical abuse (ref) | 906 | 1.07 (0.94-1.21) | 1.03 (0.91-1.16)^a^ | 1.02 (0.90-1.15) |
| Physical abuse and distress vs psychological abuse (ref) | 1201 | 1.00 (0.81-1.23) | 1.02 (0.84-1.24)^a^ | 1.02 (0.84-1.24) |
| All three traumatic experiences vs psychological abuse and physical abuse only (ref)^j^ | 526 | 1.05 (0.93-1.19) | 1.05 (0.93-1.19) | 1.07 (0.94-1.20) |
| All three traumatic experiences vs psychological abuse and substance abuse distress only (ref)^k^ | 239 | 1.01 (0.87-1.18) | 1.03 (0.88-1.20) | 1.02 (0.87-1.19) |
| All three traumatic experiences vs physical abuse and substance abuse distress only (ref)^l^ | 177 | 1.08 (0.86-1.36) | 1.06 (0.85-1.33) | 1.08 (0.85-1.36) |
|  |  | **Well-being (SWLS)** | | |
| Trauma frequency (reference: not exposed)^f^ | 10907 | Ref | Ref ^a^ | Ref |
| - Exposed to any one traumatic experience | 525 | **1.19 (1.13-1.26)** | **1.16 (1.10-1.23)** | **1.14 (1.08-1.20)** |
| - Exposed to any two traumatic experiences | 1416 | **1.21 (1.17-1.25)** | **1.17 (1.13-1.22)** | **1.14 (1.10-1.18)** |
| - Exposed to all three traumatic experiences | 133 | **1.25 (1.14-1.37)** | **1.17 (1.07-1.29)** | **1.14 (1.03-1.25)** |
| Psychological abuse vs physical abuse (ref) | 1431 | 0.98 (0.92-1.04) | 0.98 (0.92-1.04)^d^ | 0.99 (0.93-1.05)^i^ |
| Psychological abuse vs substance abuse distress (ref) | 1844 | 1.04 (0.98-1.10) | 1.02 (0.97-1.08)^c^ | 1.02 (0.96-1.07)^h^ |
| Physical abuse vs substance abuse distress (ref) | 1549 | **1.06 (1.00-1.13)** | 1.03 (0.97-1.10)^b^ | 1.03 (0.96-1.09)^g^ |
| Psychological abuse and physical abuse vs substance abuse distress (ref) | 1319 | **1.07 (1.01-1.15)** | 1.07 (1.00-1.14)^a^ | 1.05 (0.98-1.12) |
| Psychological abuse and distress vs physical abuse (ref) | 906 | 1.09 (0.99-1.19) | 1.09 (0.99-1.20)^a^ | 1.08 (0.98-1.18) |
| Physical abuse and distress vs psychological abuse (ref) | 1201 | 1.01 (0.85-1.19) | 1.04 (0.89-1.22)^a^ | 1.05 (0.90-1.23) |
| All three traumatic experiences vs psychological abuse and physical abuse only (ref)^j^ | 526 | 0.98 (0.88-1.10) | 0.98 (0.88-1.09) | 0.99 (0.88-1.10) |
| All three traumatic experiences vs psychological abuse and substance abuse distress only (ref)^k^ | 239 | 0.92 (0.81-1.04) | 0.90 (0.79-1.03) | 0.91 (0.80-1.04) |
| All three traumatic experiences vs physical abuse and substance abuse distress only (ref)^l^ | 177 | 1.00 (0.83-1.21) | 0.91 (0.75-1.10) | 0.92 (0.75-1.12) |

All significant associations (*p*<0.05) are in bold.

^a^Adjusted for confounding variables.

^b^Adjusted for confounding variables and psychological abuse.

^c^Adjusted for confounding variables and physical abuse.

^d^Adjusted for confounding variables and substance abuse distress.

^e^Adjusted for confounding variables and mediators.

fTest for linear trend *p*<0.05

^g^Adjusted for confounding variables, mediators and psychological abuse.

^h^Adjusted for confounding variables, mediators and physical abuse.

^i^Adjusted for confounding variables, mediators and substance abuse distress.

^j^Ps_1_Ph_1_D_1_ vs Ps_1_Ph_1_D_0_ (ref)

^k^Ps_1_Ph_1_D_1_ vs Ps_1_Ph_0_D_1_ (ref)

^l^Ps_1_Ph_1_D_1_ vs Ps_0_Ph_1_D_1_ (ref)

EQ-5D: Health was assessed by the EQ-5D generic measure of health-related quality of life

SWLS: Well-being was measured by the satisfaction with life scale (SWLS)

**Table S7. Effect of exposure to traumatic experiences in childhood on health (EQ-5D), and subjective well-being (SWLS).**

|  | Crude effects | | Total effects | | Direct effects | | Proportion mediated (Indirect effects) |
| --- | --- | --- | --- | --- | --- | --- | --- |
| Childhood  Trauma combinations | Unadjusted | | Adjusted for confounding variables ^c^ | | Adjusted for confounding variables^c^ and mediators^a,b^ | |  |
|  | RR (95% CI) | % explained^e^ | RR (95% CI) | % explained^e^ | RR (95% CI) | % explained^e^ | %attentuation^d^ (95% CI) |
|  | **Health (EQ-5D) n=9312** | | |  |  |  |  |
| No trauma (Ps_0_Ph_0_D_0_) | 1.00 | Ref | 1.00 | Ref | 1.00 | Ref | Ref |
| Psychological abuse only (Ps_1_Ph_0_D_0_) | **1.19(1.12-1.28)** | 28.88 | **1.24(1.15-1.34)** | 22.59 | **1.22(1.13-1.32)** | 26.60 | 6.74(-1.25-16.44) |
| Physical abuse only (Ps_0_Ph_1_D_0_) | **1.12(1.00-1.26)** | 6.47 | **1.15(1.01-1.30)** | 3.92 | 1.12(0.99-1.26) | 4.18 | 19.59(-0.94-111.61) |
| Substance abuse distress only (Ps_0_Ph_0_D_1_) | 1.05(0.98-1.13) | 3.81 | 1.02(0.94-1.11) | 0.22 | 1.00(0.93-1.09) | 0.74 | **84.80(18.01-54454.66)** |
| Psychological and physical abuse (Ps_1_Ph_1_D_0_) | **1.29(1.21-1.38)** | 33.24 | **1.35(1.25-1.47)** | 40.40 | **1.27(1.18-1.38)** | 36.05 | **22.48(14.96-34.27)** |
| Psychological abuse and substance abuse distress (Ps_1_Ph_0_D_1_) | **1.34(1.18-1.51)** | 10.53 | **1.30(1.12-1.51)** | 7.53 | **1.23(1.06-1.42)** | 6.19 | 23.52(-6.46-65.32) |
| Physical abuse and substance abuse distress (Ps_0_Ph_1_D_1_) | **1.25(1.02-1.55)** | 3.22 | **1.32(1.01-1.71)** | 3.51 | **1.29(1.00-1.67)** | 2.66 | 9.27(-25.61-61.99) |
| Psychological abuse, physical abuse and substance abuse distress (Ps_1_Ph_1_D_1_) | **1.37(1.23-1.52)** | 14.84 | **1.44(1.29-1.61)** | 21.82 | **1.38(1.24-1.54)** | 23.58 | **12.72(2.77-23.34)** |
|  | **Well-being (SWLS) n=8965** | | | | | | |
| No trauma (Ps_0_Ph_0_D_0_) | 1.00 | Ref | 1.00 | Ref | 1.00 | Ref | Ref |
| Psychological abuse only (Ps_1_Ph_0_D_0_) | **1.20(1.14-1.27)** | 22.68 | **1.18(1.11-1.25)** | 23.13 | **1.15(1.09-1.23)** | 22.06 | **12.20 (1.85-24.72)** |
| Physical abuse only (Ps_0_Ph_1_D_0_) | **1.26(1.17-1.36)** | 13.33 | **1.23(1.13-1.34)** | 15.95 | **1.20(1.11-1.31)** | 20.79 | 10.94(-0.51-26.50) |
| Substance abuse distress only (Ps_0_Ph_0_D_1_) | **1.14(1.08-1.21)** | 17.86 | **1.13(1.07-1.20)** | 17.39 | **1.11(1.05-1.18)** | 12.76 | **16.67(5.66-36.44)** |
| Psychological and physical abuse (Ps_1_Ph_1_D_0_) | **1.28(1.21-1.35)** | 25.73 | **1.23(1.15-1.31)** | 24.79 | **1.15(1.08-1.23)** | 20.76 | **31.96(20.50-49.52)** |
| Psychological abuse and substance abuse distress (Ps_1_Ph_0_D_1_) | **1.37(1.26-1.50)** | 9.78 | **1.33(1.20-1.48)** | 9.74 | **1.26(1.14-1.40)** | 12.87 | **20.76(5.44-39.53)** |
| Physical abuse and substance abuse distress (Ps_0_Ph_1_D_1_) | **1.25(1.06-1.48)** | 2.62 | **1.27(1.07-1.52)** | 3.24 | **1.25(1.04-1.50)** | 4.51 | 8.78(-16.65-47.73) |
| Psychological abuse, physical abuse and substance abuse distress (Ps_1_Ph_1_D_1_) | **1.25(1.14-1.38)** | 8.02 | **1.20(1.08-1.33)** | 5.76 | **1.16(1.04-1.28)** | 6.24 | **20.74(2.19-55.21)** |

Ps_0_Ph_0_D_0_: Not exposed to psychological abuse, physical abuse and substance abuse distress in childhood. Ps_1_Ph_0_D_0_: Exposed to psychological abuse but not physical abuse and substance abuse distress. Ps_0_Ph_1_D_0_: Exposed to physical abuse but not psychological abuse and substance abuse distress. Ps_0_Ph_0_D_1_: Exposed to substance abuse distress, but not psychological abuse and physical abuse. Ps_1_Ph_1_D_0_: Exposed to both psychological and physical abuse but not substance abuse distress. Ps_1_Ph_0_D_1_: Exposed to both psychological abuse and substance abuse distress but not physical abuse. Ps_0_Ph_1_D_1_: Exposed to both physical abuse and substance abuse distress but not psychological abuse. Ps_1_Ph_1_D_1_: Exposed to psychological abuse, physical abuse, and substance abuse distress.

^a^Social support factors were measured by instrumental and emotional support. Instrumental support: Do you have enough friends who can give you help and support when you need it? (yes, no); Emotional support: Do you have enough friends you can talk confidentially with? (yes, no)

^b^Behavioural factors were measured by two questions: Do you/did you smoke daily? (yes, now; yes, previously; never); How many units of alcohol (a beer, a glass of wine or a drink) do you usually drink when you drink alcohol? (1-4, 5-6, 7-9, 10 or more).

^c^Confounding variables were age, gender, fathers’ education, mothers’ education and childhood financial conditions.

^d^ The percentages show the reduction in relative risk (RR) in model adjusted for mediators, compared to model adjusted only for confounding variables. For instance, the reduction in the RR for Well-being for the Ps_1_Ph_0_D_0_ group when including mediators to the first model, is [(1.176261-1.154754)/(1.176261-1.00)]*100 = 12.20%.

^e^ Shapley decomposition of dissimilarity index. Percentage explained by each combination of traumatic experience.

All significant associations (*p*<0.05) are in **bold.**

EQ-5D: Health was assessed by the EQ-5D generic measure of health-related quality of life

SWLS: Well-being was measured by the satisfaction with life scale (SWLS)

**Table S8. Effect of traumatic experience in childhood on health (EQ-5D), and subjective well-being (SWLS) in imputed dataset with multiple imputation (n=12,981)**

|  | Crude effects | Total effects | Direct effects | Indirect effects |
| --- | --- | --- | --- | --- |
|  | Unadjusted | Adjusted for confounding variables^c^ | Adjusted for confounding variables^c^ and mediators^a,b^ | Proportion mediated^±^ |
|  | RR (95% CI) | RR (95% CI) | RR (95% CI) | %attentuation^d^ (95% CI) |
|  | **Health (EQ-5D)** | | | |
| No trauma (Ps_0_Ph_0_D_0_) | 1.00 (ref) | 1.00 (ref) | 1.00 (ref) | Ref |
| Psychological abuse only (Ps_1_Ph_0_D_0_) | **1.17 (1.09-1.25)** | **1.22 (1.16-1.24)** | **1.19 (1.15-1.21)** | **9.12 (5.41-14.20)** |
| Physical abuse only (Ps_0_Ph_1_D_0_) | 1.10 (0.99-1.22) | **1.16 (1.12-1.27)** | **1.13 (1.05-1.22)** | **19.90 (10.94-34.29)** |
| Substance abuse distress only (Ps_0_Ph_0_D_1_) | 1.03 (0.96-1.10) | 1.04 (1.00-1.07) | 1.02 (0.98-1.05) | 42.32 (-43.66-248.44) |
| Psychological and physical abuse (Ps_1_Ph_1_D_0_) | **1.27 (1.18-1.35)** | **1.33 (1.27-1.38)** | **1.26 (1.22-1.31)** | **18.29 (15.59-19.38)** |
| Psychological abuse and substance abuse distress (Ps_1_Ph_0_D_1_) | **1.31 (1.16-1.48)** | **1.33 (1.27-1.60)** | **1.27 (1.21-1.49)** | **15.51 (9.21-23.98)** |
| Physical abuse and substance abuse distress (Ps_0_Ph_1_D_1_) | **1.23 (1.00-1.51)** | **1.33 (1.14-1.54)** | **1.30 (1.14-1.44)** | **9.17 (0.19-14.49)** |
| Psychological abuse, physical abuse and substance abuse distress (Ps_1_Ph_1_D_1_) | **1.33 (1.20-1.47)** | **1.37 (1.30-1.44)** | **1.32 (1.25-1.38)** | **12.47 (6.43-17.67)** |
|  | **Well-being (SWLS)** | | | |
| No trauma (Ps_0_Ph_0_D_0_) | 1.00 (ref) | 1.00 (ref) | 1.00 (ref) | Ref |
| Psychological abuse only (Ps_1_Ph_0_D_0_) | **1.19 (1.13-1.26)** | **1.20 (1.11-1.32)** | **1.17 (1.09-1.19)** | **16.17 (13.37-28.70)** |
| Physical abuse only (Ps_0_Ph_1_D_0_) | **1.23 (1.14-1.33)** | **1.20 (1.11-1.30)** | **1.17 (1.09-1.28)** | **14.17 (3.59-20.07)** |
| Substance abuse distress only (Ps_0_Ph_0_D_1_) | **1.13 (1.07-1.20)** | **1.10 (1.07-1.15)** | **1.08 (1.05-1.12)** | **18.02 (12.53-23.11)** |
| Psychological and physical abuse (Ps_1_Ph_1_D_0_) | **1.27 (1.20-1.34)** | **1.22 (1.18-1.27)** | **1.16 (1.12-1.20)** | **27.57 (25.23-30.73)** |
| Psychological abuse and substance abuse distress (Ps_1_Ph_0_D_1_) | **1.36 (1.25-1.49)** | **1.31 (1.27-1.49)** | **1.26 (1.18-1.39)** | **14.82 (9.33-20.74)** |
| Physical abuse and substance abuse distress (Ps_0_Ph_1_D_1_) | **1.25 (1.06-1.47)** | **1.24 (1.12-1.30)** | **1.23 (1.13-1.29)** | 6.27 (-8.92-64.18) |
| Psychological abuse, physical abuse and substance abuse distress (Ps_1_Ph_1_D_1_) | **1.25 (1.14-1.37)** | **1.17 (1.10-1.23)** | **1.13 (1.06-1.19)** | **22.29 (17.14-38.37)** |

Ps_0_Ph_0_D_0_: Not exposed to psychological abuse, physical abuse and substance abuse distress in childhood. Ps_1_Ph_0_D_0_: Exposed to psychological abuse but not physical abuse and substance abuse distress. Ps_0_Ph_1_D_0_: Exposed to physical abuse but not psychological abuse and substance abuse distress. Ps_0_Ph_0_D_1_: Exposed to substance abuse distress, but not psychological abuse and physical abuse. Ps_1_Ph_1_D_0_: Exposed to both psychological and physical abuse but not substance abuse distress. Ps_1_Ph_0_D_1_: Exposed to both psychological abuse and substance abuse distress but not physical abuse. Ps_0_Ph_1_D_1_: Exposed to both physical abuse and substance abuse distress but not psychological abuse. Ps_1_Ph_1_D_1_: Exposed to psychological abuse, physical abuse, and substance abuse distress.

^a^ Social support factors were measured by instrumental and emotional support. Instrumental support: Do you have enough friends who can give you help and support when you need it? (yes, no); Emotional support: Do you have enough friends you can talk confidentially with? (yes, no)

^b^Behavioural factors were measured by two questions: Do you/did you smoke daily? (yes, now; yes, previously; never); How many units of alcohol (a beer, a glass of wine or a drink) do you usually drink when you drink alcohol? (1-4, 5-6, 7-9, 10 or more).

^c^Confounding variables were age, gender, fathers’ education, mothers’ education and childhood financial conditions.

^±^ The percentages show the reduction in relative risk (RR) in model adjusted for mediators, compared to model adjusted only for confounding variables. For instance, the reduction in the RR for EQ-5D for the Ps_1_Ph_0_D_0_ group when including mediators to the first model, is [(1.215-1.194)/(1. 1.215-1.00)]*100 = 9.119%.

EQ-5D: Health was assessed by the EQ-5D generic measure of health-related quality of life

SWLS: Well-being was measured by the satisfaction with life scale (SWLS)

All significant associations (*p*<0.01) are in **bold**.

REFERENCES

Barros, A., & Hirakata, V. (2003). Alternatives for logistic regression in cross-sectional studies: an empirical comparison of models that directly estimate the prevalence ratio. *BMC Medical Research Methodology, 3*(1), 21.

Diener, E., Emmons, R. A., Larsen, R. J., Griffin, S. (1985). The Satisfaction with Life Scale. *Journal of Personality Assessment, 49*, 71-75.

Greenacre, M. (2007). *Correspondence Analysis in Practice, Second Edition*: CRC Press.

Greenacre, M. (2010). *Biplots in Practice*: Fundación BBVA (FBBVA).

Oishi, S. (2006). The concept of life satisfaction across cultures: An IRT analysis. *Journal of Research in Personality, 40*(4), 411-423. doi:<http://dx.doi.org/10.1016/j.jrp.2005.02.002>

Sheikh, M. A. (2014). Parental income is more important than parental education to children’s health and wellbeing in adulthood: Evidence from The Tromsø Study. *Occupational and Environmental Medicine, 71*(Suppl 1), A119. doi:10.1136/oemed-2014-102362.374

Sheikh, M. A. (2015). Childhood trauma and adult health and wellbeing. *The European Journal of Public Health, 25*(suppl 3). doi:10.1093/eurpub/ckv176.301

Sheikh, M. A., Abelsen, B., & Olsen, J. A. (2014). Role of respondents' education as a mediator and moderator in the association between childhood socio-economic status and later health and wellbeing. *BMC Public Health, 14*(1), 1172.

The EuroQol Group. (1990). EuroQol--a new facility for the measurement of health-related quality of life. The EuroQol Group. *Health Policy, 16*(3), 199-208.

Zou, G. (2004). A Modified Poisson Regression Approach to Prospective Studies with Binary Data. *American Journal of Epidemiology, 159*(7), 702-706. doi:10.1093/aje/kwh090
